# Building the sugarcane genome for biotechnology and identifying evolutionary trends

**DOI:** 10.1186/1471-2164-15-540

**Published:** 2014-06-30

**Authors:** Nathalia de Setta, Cláudia Barros Monteiro-Vitorello, Cushla Jane Metcalfe, Guilherme Marcelo Queiroga Cruz, Luiz Eduardo Del Bem, Renato Vicentini, Fábio Tebaldi Silveira Nogueira, Roberta Alvares Campos, Sideny Lima Nunes, Paula Cristina Gasperazzo Turrini, Andreia Prata Vieira, Edgar Andrés Ochoa Cruz, Tatiana Caroline Silveira Corrêa, Carlos Takeshi Hotta, Alessandro de Mello Varani, Sonia Vautrin, Adilson Silva da Trindade, Mariane de Mendonça Vilela, Carolina Gimiliani Lembke, Paloma Mieko Sato, Rodrigo Fandino de Andrade, Milton Yutaka Nishiyama, Claudio Benicio Cardoso-Silva, Katia Castanho Scortecci, Antônio Augusto Franco Garcia, Monalisa Sampaio Carneiro, Changsoo Kim, Andrew H Paterson, Hélène Bergès, Angélique D’Hont, Anete Pereira de Souza, Glaucia Mendes Souza, Michel Vincentz, João Paulo Kitajima, Marie-Anne Van Sluys

**Affiliations:** Departamento de Botânica – Instituto de Biociências, Universidade de São Paulo, Rua do Matão 277, São Paulo, 05508-090 SP Brazil; Universidade Federal do ABC, Rua Santa Adélia, 166, Santo André, 09210-170 Brazil; Escola Superior de Agricultura Luiz de Queiroz, Departamento de Genética, Universidade de São Paulo, Av. Padua Dias, 11, Agronomia, 13418-900 Piracicaba, SP Brasil; Centro de Biologia Molecular e Engenharia Genética, Universidade Estadual de Campinas, Av. Cândido Rondon, 400, 13083-875 Campinas, Brazil; Departamento de Genética, Instituto de Biociências, Universidade Estadual Paulista, campus de Botucatu, Distrito de Rubião Jr., s/n, 18618-000 Botucatu, Brazil; Departamento de Bioquímica, Instituto de Química, Av. Prof. Lineu Prestes, 748, São Paulo, 05508-900 SP Brazil; INRA – CNRGV, 24 Chemin de Borde Rouge, Auzeville, CS 52627, 31326 Castanet Tolosan Cedex, France; Departamento de Biologia Celular e Genética – UFRN, Campus Universitário s/n, Natal, RN 59072-970 Brazil; Centro de Ciências Agrárias, Universidade Federal de São Carlos, Araras, SP Brazil; Departments of Plant Biology, Crop and Soil Science, and Genetics, University of Georgia, 111 Riverbend Rd, Athens, GA 30602 USA; CIRAD, UMR1096, TA40/03 Avenue Agropolis, 34398 Montpellier Cedex 5, France; Mendelics Genomic Analysis, Rua Cubatão 86, São Paulo, SP Brazil

**Keywords:** *Saccharum*, Bacterial artificial chromosome sequencing, Polyploidy, Genome, Genetics, Grasses

## Abstract

**Background:**

Sugarcane is the source of sugar in all tropical and subtropical countries and is becoming increasingly important for bio-based fuels. However, its large (10 Gb), polyploid, complex genome has hindered genome based breeding efforts. Here we release the largest and most diverse set of sugarcane genome sequences to date, as part of an on-going initiative to provide a sugarcane genomic information resource, with the ultimate goal of producing a gold standard genome.

**Results:**

Three hundred and seventeen chiefly euchromatic BACs were sequenced. A reference set of one thousand four hundred manually-annotated protein-coding genes was generated. A small RNA collection and a RNA-seq library were used to explore expression patterns and the sRNA landscape. In the sucrose and starch metabolism pathway, 16 non-redundant enzyme-encoding genes were identified. One of the sucrose pathway genes, sucrose-6-phosphate phosphohydrolase, is duplicated in sugarcane and sorghum, but not in rice and maize. A diversity analysis of the *s6pp* duplication region revealed haplotype-structured sequence composition. Examination of hom(e)ologous loci indicate both sequence structural and sRNA landscape variation. A synteny analysis shows that the sugarcane genome has expanded relative to the sorghum genome, largely due to the presence of transposable elements and uncharacterized intergenic and intronic sequences.

**Conclusion:**

This release of sugarcane genomic sequences will advance our understanding of sugarcane genetics and contribute to the development of molecular tools for breeding purposes and gene discovery.

**Electronic supplementary material:**

The online version of this article (doi:10.1186/1471-2164-15-540) contains supplementary material, which is available to authorized users.

## Background

Sugarcane is an important crop worldwide, producing 80% of the world’s raw sugar and is increasingly used for bio-fuel [1]. A key goal in meeting growing demand is to improve sugarcane yield and accelerate selection for desirable traits. Genomics has been shown to be successful in genome-assisted breeding programs for selecting superior genotypes and more efficient breeding strategies.

Species of the *Saccharum* complex (sugarcane) are part of the Poaceae family and together with *Sorghum*, *Zea* and other genera comprise the Panicoidae superfamily, one of the C4 photosynthetic grass lineages (Additional file 1: Figure S1) [2]. At the end of nineteenth century, early sugarcane breeders in Java and India carried out crosses between *S. officinarum* and *S. spontaneum* in order to introduce vigor and resistance genes from wild *S. spontaneum*, while quickly recovering the high sugar content of *S. officinarum* cultivars [3]. Modern sugarcane cultivars are derived from those early interspecific genotypes, followed by several cycles of intercrossing and selection. They are polyploid aneuploid hybrids with unequal contribution from *S. officinarum* (80–90%) and *S. spontaneum* (10–20%) parental genomes and a small percentage of recombinant chromosomes [4, 5]. Sugarcane hybrids have ploidy levels of 10 or more and have a much larger total genome size (R570 cultivar, 10,000 Mb and 2n = 115) than that of maize (5500 Mb, 2n = 20), sorghum (1600 Mb, 2n = 20) or rice (860 Mb, 2n = 24) reflecting the high polyploidy level of sugarcane cultivars [6].

The sorghum genome, the closest related fully sequenced and annotated genome to sugarcane, is widely recognized as reference genome for comparative analysis. The origin of modern sugarcane cultivars raises issues not only related to the extent and nature of the divergence of the sugarcane and sorghum genomes, but also about the relationships (meiosis and expression dosage) among hom(e)ologous loci. Equally importantly, deciphering the sugarcane genome is a major goal for improving genome wide assisted selection breeding opportunities worldwide. However, the hybrid polyploid nature of modern cultivars imposes limitations to breeders in understanding genotype to phenotype allelic variation and dosage. The present study was undertaken within the framework of a larger sequencing initiative to generate a comprehensive dataset, providing information on sugarcane genome structure and function as a basis for future functional genetic studies.

## Results

### BAC sequencing and repeat annotation

Three hundred and seventeen sugarcane bacterial artificial chromosome (BAC) inserts of a R570 cultivar genomic library [7] were sequenced. A total of 189 BACs were selected using probes homologous to 84 previously described expressed genes [8]. Seventy-eight BACs were selected for using probes based on five superfamilies of transcriptionally active transposable elements (TEs). The remaining 50 BACs were selected in a previous study using RFLP markers from nine sugarcane linkage groups [7] (Additional file 2: Table S1).

In total, 36.58 million bases were sequenced with an average of 361 bp per read, 25,000 reads per BAC, and 92 X coverage (Additional file 2: Table S1). This represents 3.7% of the monoploid complement, based on the estimate of a 10 Gb genome size for the decaploid hybrid cultivar R570 [6]. Two hundred and five BACs were assembled into one contig each and the remaining 112 BACs were assembled into an average of 3.15 contigs. Although not all BACs were single contigs, all have a proposed scaffold and are a single-fasta file. To date, most of the gaps have repetitive sequences at the ends. The minimum, maximum, median, and average sizes of BAC assemblies were 12.25, 259.2, 115.38 and 112.34 Kb, respectively. A BLASTn search indicates that none of the sequences were derived from chloroplast or mitochondrial genomes.

The repetitive content was estimated from BAC assemblies using the Repbase database [9] and a curated sugarcane Long Terminal Repeat (LTR) retrotransposon database [10]. Fifty percent of the BAC sequences are repetitive, 49.4% transposable elements (TEs) and 0.43% satellite repeats (Additional file 3: Table S2 and Additional file 4: Table S3). Of the TEs, LTR retrotransposons are the most abundant (40.86%), followed by DNA transposons (7.93%), and non-LTR retrotransposons. TE content of individual BACs is highly heterogeneous, varying from zero (a ribosomal DNA BAC) to 98.7%. Miniature inverted-repeat TEs (MITEs) represent 3% of the sequences. Of 3,663 curated MITEs, the most abundant types are *Tourist* (63.8%), followed by *Stowaway* (27.9%), *hAT* (5.6%), *MULE* (1.7%), unclassified (0.6%) and *CACTA* (0.4%). Sugarcane has a ratio of *Gypsy*-*Ty3* to *Copia*-*Ty1* elements (1.3 to 1) more closely resembling that of maize (1.6 to 1), than of sorghum (3.7 to 1) or rice (4.9 to 1) genomes, suggesting a closer correlation with genome size rather than with phylogeny.

### Gene annotation and CDS validation

BAC assemblies masked for repetitive sequences were analyzed by a combination of de novo gene prediction software programs and searches against databases to identify non-TE coding genes. For 14 BACs there were no predicted protein-coding genes identified. A total of 1,400 coding regions were predicted and annotated. An average of 3.8 CDSs (Coding DNA Sequences) were found per 100 Kb, representing one protein-coding gene per 26.12 Kb. RNA-seq data from the cultivar RB92-5345 and the sugarcane assembled EST sequences (SASs) from the SUCEST (sugarcane EST) Project [8] were used to validate CDSs. All CDSs mapped against at least one SAS and 1,218 mapped against at least one pair of RNA-seq reads (87% of the total). This may be because there was no detectable expression of these genes under the experimental conditions used, or high sequence divergence between the two cultivars (R570 and RB92-5345) for these specific loci, and/or false positive gene annotation.

Using Blast2GO, GO (gene ontology) terms were assigned to 1,081 of the 1,400 predicted protein-coding sequences (77.8%). A total of 4,730 GO functional terms were assigned to the 1,081 sequences. GO terms were placed into three broad categories, Biological Process 1,884 (39.8%), Molecular Function 1,502 (31.7%) and Cellular Component 1,344 (28.5%). The most abundant terms in the Biological Process category include cellular process and metabolic process and in the Molecular Function category, catalytic activity and binding. In the Cellular Component category, the most common terms were organelle, membrane and cell (Additional file 5: Figure S2). BLASTp searches against the NCBI nr database [11] confirmed that most of the sugarcane protein sequences are most similar to those of sorghum (Additional file 6: Figure S3). The top BLAST match for 908 protein sequences was to sorghum sequences.

CDSs were broadly distributed amongst the 17 functional categories described by the SUCEST Project [8] (Additional file 7: Figure S4). Transcriptionally active genes (as determined by SUCEST) were evaluated by a WU-blast search using SASs as queries against the BACs. Sixteen ½ percent of the SASs matched the unmasked BACs, i.e. for 83.5% of the SASs there was no match to the unmasked BACs. In the masked BACS, there were matches for 13% of the SASs. These percentages may represent an overestimation due to multiple matches to hom(e)ologous or paralogous genes. Annotated TEs were homologous to 3.5% of SASs, suggesting that 3.5% of the transcriptome is derived from TEs. Our present estimate is close to that of a previous estimate of 2.3% [8].

### Metabolic pathway genes

Mapping of annotated CDSs and SASs using the KEGG Mapper tool at MG-RAST [12] provided a global view of known sugarcane metabolic pathways. The comparison between BAC CDSs and SASs mapping identified genes not previously reported in the sugarcane transcriptome. EC numbers were assigned to 803 predicted enzyme-coding genes distributed amongst various metabolic pathways, including those involved in carbohydrate, lipid and amino acid metabolism (Additional file 8: Figure S5). Most of the predicted enzymes (594) were identified in the SASs collection only, 122 were common to the SAS and BAC sequences and 66 identified by BAC sequence alone. Genes predicted from BAC sequence alone included enzyme-coding genes from the carotenoid, amino acid, diterpenoids and other fatty acids biosynthesis pathways (Additional file 9: Table S4).

**Figure 1:**
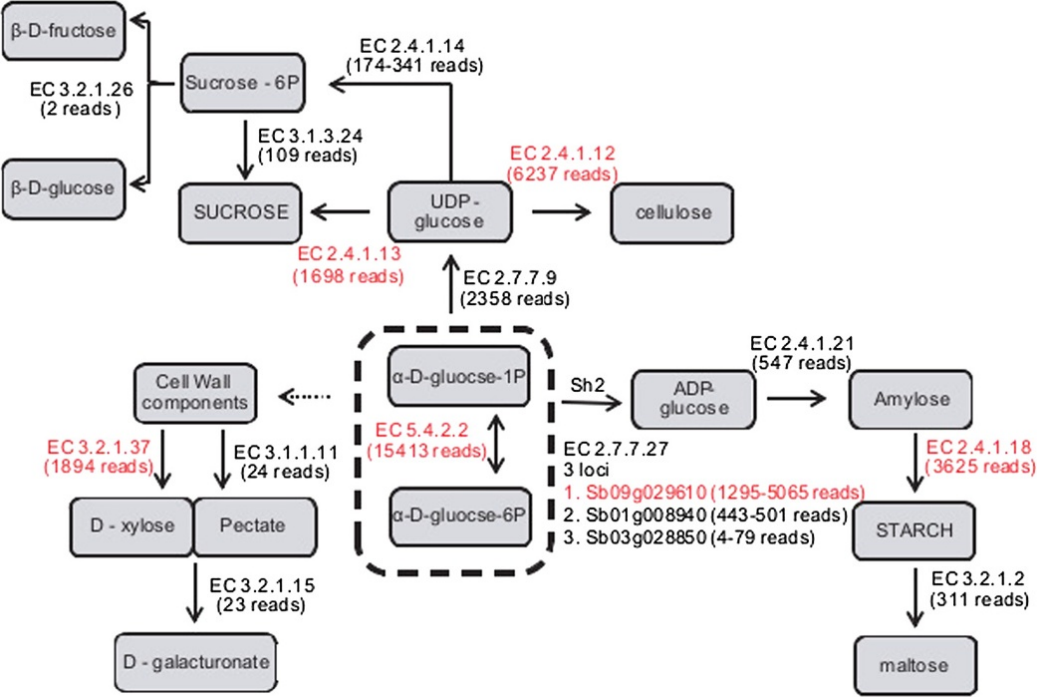
**Schematic representation of the sucrose, cellulose and starch metabolic pathways, showing genes identified with supporting RNA-seq mapping information.** The grey boxes represent enzyme products. The arrows represent enzyme reactions, solid arrows are enzyme reactions where the predicted enzyme-coding genes were identified in sugarcane, dotted arrows where the gene was not identified. EC numbers are shown for the predicted enzyme-coding genes identified. EC numbers in red indicate predicted enzyme-coding genes that were mapped with more than a thousand RNA-seq reads. The number of mRNA reads mapped is indicated in parentheses below the EC number. If more than one BAC to a single Sorghum loci was sequenced, the minimum and the maximum number of reads mapped to all BACs are shown. EC 3.2.1.26: beta-fructofuranosidase, 3.1.3.24: sucrose-6-phosphate phosphohydrolase, EC 2.4.1.14: sucrose phosphate synthase, EC 2.4.1.12: cellulose synthase, EC 2.4.1.13: sucrose synthase, EC 2.7.7.9: UDP glucose pyrophosphorylase, EC 3.2.1.37: xylan 1,4-beta-xylosidase, EC 3.1.1.11: pectinesterase, EC 3.2.1.15: polygalacturonase, EC 5.4.2.2: phosphoglucomutase, EC 2.7.7.27: glucose-1-phosphate adenylyltransferase, EC 2.4.1.21: starch synthase, EC 2.4.1.18: 1,4-alpha-glucan branching enzyme and EC 3.2.1.2: beta-amylase.

Twenty-nine genes involved in sucrose and starch metabolism (Additional file 10: Table S5) were identified, representing 16 non-redundant genes. Figure 1 is a schematic representation of the central carbon metabolism pathway where phosphoglucomutase, responsible for the reversible conversion of α-D-glucose-1P and α-D-glucose-6P, is at the center of the diagram. These two enzymes, together with β-D-fructose-6P, are key components in the balancing of metabolic activity in terms of source (sucrose), sink (starch) and growth (cell wall components). Genes encoding the enzymatic components of cellulose (cellulose synthase) and sucrose synthesis (sucrose synthase and sucrose phosphate phosphatase) were also identified.RNA-seq data from 5-day old germinating lateral buds of the RB92-5345 cultivar was mapped to the 16 non-redundant enzyme-coding genes involved in sucrose and starch metabolism. If more than one hom(e)ologous loci to a single Sorghum loci was identified, the minimum and the maximum number of reads mapped to all loci are shown (Figure 1). The high number of mapped RNA-seq suggests that it is an active pathway involved in growth of the young bud tissue. The most actively transcribed gene encodes phosphoglucomutase followed by cellulose synthase and glucose-1-phosphate adenylyltransferase. Sucrose synthesis is mainly driven by the activity of sucrose synthase, however the two-step process catalyzed by sucrose phosphate synthase and sucrose-6-phosphate phosphohydrolase is also transcribed.

Three loci were identified for the glucose-1-phosphate adenylyltransferase gene, the enzyme that catalyzes the conversion of α-D-glucose-1-phosphate into ADP-glucose. This is the first and key regulatory step in starch synthesis [13]. Based on our RNA-seq mapping data, one locus was more highly expressed than other two (Figure 1). The glucose-1-phosphate adenylyltransferase enzyme is composed of two large and two small subunits [13]. In maize, the large subunit is coded by the maize *Sh2* locus, which is well characterized in plants, and in particular in grasses. Four loci are responsible for this reaction in sorghum (Sb09g029610, Sb01g008940, Sb02g020410 and Sb03g028850). Three of these loci were identified in sugarcane BACs, Sb09g029610 (SHCRBa_003_M06, SHCRBa_026_K06 and SHCRBa_078_K12), Sb01g008940 (SHCRBA_027_I16, SHCRBa_033_L20, SHCRBa_073_J10 and SHCRBa_119_J13) and Sb03g028850 (SHCRBa_009_B01, SHCRBa_012_A01 and SHCRBa_022_D05). The sugarcane orthologous of BACs SHCRBa_022_D05, SHCRBa_012_A01 and SHCRBa_009_B01 correspond to the *Sh2* locus (Figure 1).

Sucrose-6-phosphate phosphohydrolase (S6PP) catalyzes the reaction from sucrose-6P to sucrose. There is a tandem duplication of this gene, not previously published, but evidenced by genomic sequences in the Phytozome database [14], in sorghum (Sb09g003460.1 and Sb09g003463.1), *Setaria italica* (Si022142m and Si024709m) and *Panicum virgatum* (Pavirv00037112m and Pavir00037113m). However, the same duplication is not found in maize or any of the other grass genomes available. The BAC SHCRBa_104_G22 annotation suggests that *s6pp* is duplicated in the sugarcane genome. In order to better understand this region, which is important for sucrose synthesis, we examined the composition of the intergenic region between the two copies of *s6pp* in modern sugarcane cultivars, *Saccharum* species*,* sorghum and *Miscanthus* sp. by sequencing a PCR amplified 1,539 bp fragment. One hundred and ninety amplicons were aligned against sorghum sequences and the R570 BAC (SHCRBa_104_G22). Overall nucleotide identity is high, 99.988% (SD 0.001). The sorghum sequence is the most divergent with an average 99.935% (SD 0.005) identity compared with all other sequences. *S. spontaneum* sequences are more divergent from the other sugarcane sequences (99.985%, SD 0.006). Neighbor-joining and maximum likelihood phylogenetic analyses resulted in unresolved evolutionary relationships (data not shown).

**Figure 2:**
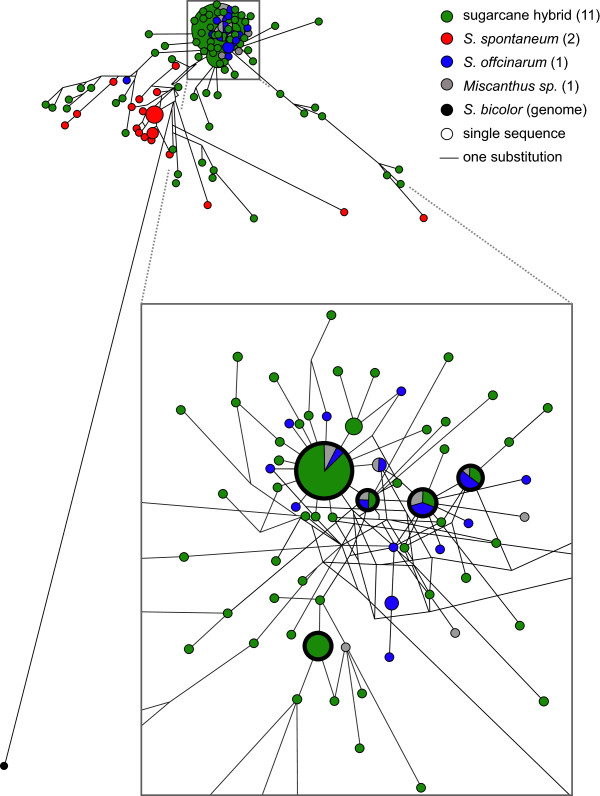
**Network analysis of the** ***s6pp*** **gene duplication region.** The network was constructed using the NETWORK 4.5.1.0 software [84] with default parameters. From a 1,539 bp alignment, 262 variable characters were used to reconstruct the network. The main figure is a closeup of part of the entire network which shown in the top left. The size of the circle is relative to the number of sequences in that haplotype. Thick bold circles represent the five main haplotypes. A single dash denotes a single substitution; the distance between clusters is therefore proportional to the number of substitutions. Numbers between parentheses in the legend show the number of cultivars analyzed.

Network analyses can be used to predict relationships amongst sequences with low diversity [15]. Because of the low sequence diversity of the sugarcane *s6pp* duplication, a haplotype network was generated to describe the relationships of sugarcane species and cultivars. Thirty percent of the sequences fell into five main haplotypes (thick bold black circlers in Figure 2). Four of these haplotypes were from *S. officinarum*, modern sugarcane hybrids and *Miscanthus* sp., the fifth is found specifically in the modern sugarcane hybrids. The most common haplotype consists of 33 sequences, from the R570 BAC (SHCRBa_104_G22), *S. officinarum*, sugarcane hybrids and *Miscanthus* sp. In networks, haplotype clustering with higher number of sequences is associated with ancestral, shared characters [16], while dispersed sequences is associated with the accumulation of nucleotide substitutions, i.e., derived characters. Although *S. spontaneum* is a parental species to modern sugarcane hybrids, *S. spontaneum* sequences are the most dispersed in the network analysis. This suggests that either particular *S. spontaneum* cultivars were used in breeding crosses or *S. spontaneum* chromosomes with the substitutions in this genomic region were not transmitted during the hybridization events.

### sRNAs in sugarcane BACs

A sRNA library from sugarcane leaf tissue [10] was mapped against the BACs to evaluate the sRNA landscape and to identify new microRNA (miRNA) genes (Additional file 11: Figure S6). This library was derived from the hybrid SP80-3280, the main cultivar used to produce the SUCEST database [8]. Most sRNAs from grasses are in the 24-nucleotide size range and therefore are most likely small interfering RNAs (siRNAs) or repeated-associated small interfering RNAs (rasiRNAs) [17]. Sixty-one percent of the sRNAs in the SP80-3280 sRNA library were in the 23–25 nt range, and 48% of them mapped to TEs identified in the BACs. The 23–25 nt RNAs mapped to the TEs are 3 × more frequent than did the smaller 20–22 nt RNAs. This pattern is expected for rasiRNAs [17] and suggests that TEs are the origin of the 23–25 nt rasiRNAs, as well as the target for sRNA-mediated gene regulation.

We searched the BACs for the 19 sugarcane primary miRNA (pri-miRNA) genes previously described [18] in an attempt to identify miRNA genes. One miRNA locus was identified. This pri-miRNA, a miR437 precursor (*SsMIR437a* gene), has high similarity (score 416) to MITE-derived hairpin sequences (DNA/Stowaway) in the hairpin region, and the intron has high similarity to a LINE/RTE non-LTR retrotransposon (score 2091, Figure 3). The high similarity between the hairpin sequence of *SsMIR437a* and a MITE corroborates recent publications positing that some plant miRNA families are derived from TEs, including MITEs [19, 20].

**Figure 3:**
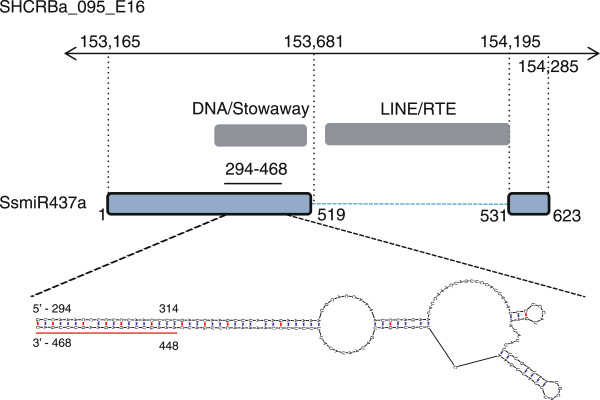
**Structure of the** ***SsMIR437a*** **gene identified in BAC SCHRBa_095_E16.** The double-arrowed solid black line shows the location within the BAC, the numbers indicate the nucleotide positions within the BAC. The number along the blue line show the position within the region. Exons are shown as blue bars, TEs as grey bars, the intron as a dashed blue line and the putative source of miRNA mature sequence as a solid red line. TEs were identified using RepeatMasker (cut-off score > 250). The miRNA mature sequence is AAAGUUAGAGAAGUUUGACUU.

### Ribosomal and pericentromeric and/or centromeric BACs

No protein-coding genes were identified in 14 BACs and these were further analysed to better understand their sequence composition. Three BACs (SCHRBa_239_N21, SCHRBa_013_I13 and SCHRBa_029_018) were predicted to be pericentromeric and/or centromeric and one (SHCRBa_039_D18) was entirely composed of ribosomal tandem repeats. The other BACs were TE rich or had no significant matches to grass protein sequences available.

The ribosomal DNA (rDNA) BAC consisted of 14 *45S* ribosomal transcription units with a portion of one unit in the reverse orientation to the other 13 (Additional file 12: Figure S7A). Each *45S* ribosomal unit was 8.8 Kb long, consisting of the *18S* (1.8 Kb) ribosomal gene, the 208 bp internal transcribed spacer 1 (ITS1), the *5.8S* (163 bp) ribosomal gene, the 216 bp internal transcribed spacer 2 (ITS2), the *26S* (3.39 Kb) ribosomal gene, and the 527 bp intergenic spacer (IGS). The *45S* ribosomal transcription units were 99.8% identical at the nucleotide level.

The three BACs classified as pericentromeric and/or centromeric contain the previously described sugarcane 137 bp centromeric repeat SCEN [21], and plant specific *Gypsy*-*Ty3* centromeric specific-like retrotransposons (*CRM*) [22]. Annotation of one of the centromeric BACs is shown in more detail in Additional file 12: Figure S7B. SCHRBa_239_N21 is 23% SCEN repeats and contains multiple copies of *CRM* and *Tat* elements. *CRM_3* was the only complete *CRM* element identified. Three *Tat* elements were identified (*Tat_2, 3* and *5*), all full-length. Four hundred and thirty nine copies of the SCEN repeat were identified with a pairwise nucleotide identity of 76.8%.

R570 cultivar metaphase spreads were examined for localization of the pericentromeric/centromeric SCHRBa_239_N21 and ribosomal BAC by FISH (Additional file 12: Figure S7C). The pericentromeric/centromeric BAC SCHRBA_239_N21 hybridized to a region consistent with it being a component of the centromeric or pericentric region of all chromosomes, however, signal strength varied among chromosomes. Additional fainter signals observed on chromosome arms were probably from non-centromeric specific LTR retrotransposons in the BAC [10]. For the ribosomal BAC, there were seven terminal, three interstitial and two undetermined signals.

### Comparative genomics with sorghum

**Figure 4:**
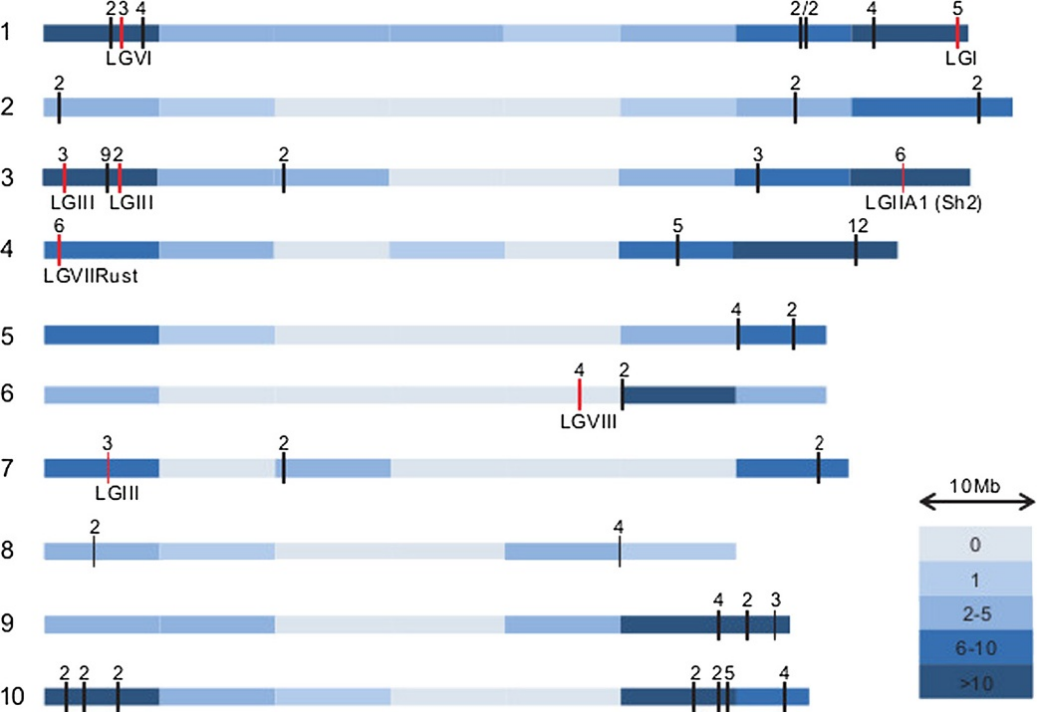
**Heatmap of the distribution of sequenced sugarcane BACs on sorghum chromosomes.** The depth of the blue colour indicates the number of BACs localized per 10 Mb. Horizontal red lines show the location of BACs selected using probes based on eight linkage groups [7]. Horizontal black lines show the location of BACs that overlap with at least one gene. Numbers above the black bars indicate the number of BACs that overlap at that point.

A BLAST based pipeline against the sorghum genome and protein databases was used to determine the distribution of the BACs relative to the sorghum genome in order to examine synteny between genomes and to gain insights into the evolution of sugarcane genomic structure [14]. A sorghum ortholog was assigned to 1,367 sugarcane predicted genes, with a redundancy of 31.6%. After excluding redundant genes (i.e. those with the same sorghum ortholog), 935 genes were analysed. For 318 genes there was a single BLAST match against sorghum-annotated proteins [14] and orthologous relationships for the remaining 617 genes were inferred by high-throughput maximum-likelihood phylogenetic analysis [23]. Using the chromosomal locations of the 935 sorghum-sugarcane orthologous, we were able to localize 265 sugarcane BACs onto sorghum chromosomal arms (Figure 4). Despite the small number of BACs and the limited number of probes, our strategy tagged all sorghum chromosome arms. The BACs mapped chiefly to the euchromatic regions, as defined by published sorghum genome annotation [24, 25]. Fifty-four BACs were not used in this mapping analysis. Fourteen of these contained no predicted protein-coding genes, among them were the pericentromeric/centromeric and ribosomal BACs described above, and 40 BACs with sorghum-sugarcane orthologous assigned to more than one sorghum chromosome.

We defined a set of stringent criteria to investigate genome size variation with this collection of sugarcane and sorghum orthologous genes. All sugarcane genes whose predicted protein coding-sequence length was not within 90% to 110% of the length of the sorghum ortholog were discarded. Only syntenic blocks with perfect colinearity of at least two genes were considered. Three hundred forty nine unique genes and 228 intergenic regions were identified within 122 syntenic blocks in 98 BACs (Additional file 13: Table S6). Although the average size of the CDSs were not significantly different, the introns and intergenic regions were statistically significantly longer in sugarcane than in sorghum (Table 1), suggesting that sugarcane underwent or is undergoing genome expansion relative to sorghum.

**Table 1:** **Comparison of sugarcane (Sc) and sorghum (Sb) genome size variation using a two-tailed Welch’s t-test**

	Average ± SD		Sc/Sb	SE	df	t-value
	Sc	Sb				
Intron length/gene (Kb)	2.22 ± 3.58	1.53 ± 1.57	1.44	0.21	476	2.87**
Intergenic length/syntenic block^a^ (Kb)	19.30 ± 25.05	11.15 ± 18.92	1.73	2.84	225	3.26**
CDS length (Kb)	1.28 ± 0.75	1.29 ± 0.75	0.99	0.06	695	0.15

**P < 0.01. a: The nucleotide length between genes in a colinear region. SE: standard error, df: degree of freedom.

The syntenic blocks were further analysed to determine the nature of the sugarcane genome expansion. These were 3.533 Mb long in total in sugarcane and 1.990 Mb in sorghum. Sugarcane and sorghum have equivalent numbers of bases encoding gene exons, 0.446 Mb and 0.449 Mb, respectively. Therefore, introns, promoters and intergenic regions may account for the sugarcane syntenic region being 1.543 Mb larger. Repeat content in sugarcane was 1.356 Mb (consisting of 1.334 Mb TEs and 0.022 Mb of single sequence repeats (SSR) and low complexity regions). Repeat content in sorghum was 0.580 Mb (TEs: 0.562 Mb; SSR and low complexity: 0.018 Mb). The difference between the two species in repeat content indicates that the 0.776 Mb expansion of the sugarcane genome is due mainly to TE amplification. Among the TE sequences, *Copia-Ty1* elements are the most common (16.17%), followed by *Gypsy-Ty3* (12.28%) and DNA transposons, including *CACTA* (5.64%), *hAT* (1.09%) and *Mutator* elements (0.19%). The large fraction of unaccounted nucleotides in both sorghum (0.961 Mb) and sugarcane (1.731 Mb) may represent unidentified novel genes, uncharacterized TEs, or as-yet-unknown genomic elements.

### Hom(e)ologous diversity and expression of *rpa1a*locus

**Figure 5:**
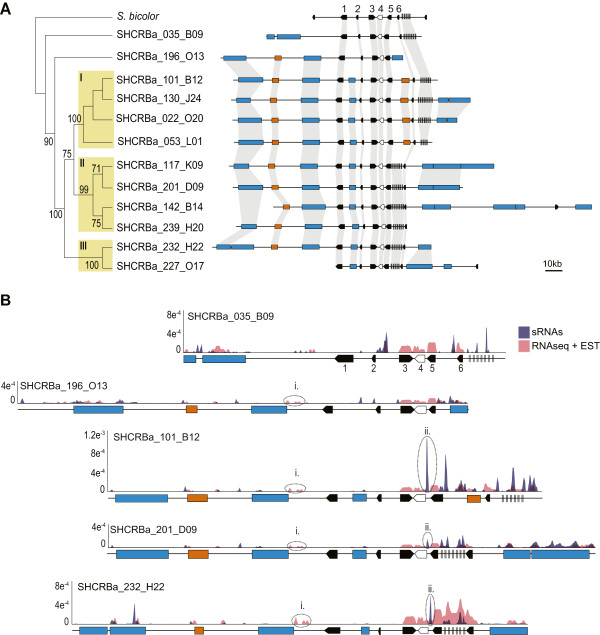
**Physical and functional relationships of** ***rpa1a*** **sugarcane hom(e)ologous BACs compared to sorghum.** The *rpa1a* genes are represented by white arrows and other genes by black arrows. LTR retrotransposons are represented by blue boxes, DNA transposons by brown boxes and *Harbinger* transposons by black vertical lines. Only contiguous TE sequences greater than 3,000 bp are shown. **A**. A physical and phylogenetic analysis of the genomic region of the *rpa1a* gene from 12 BACs and *S. bicolor*. The neighbor-joining tree was inferred with using the highest ranked substitution model (Tajima-Nei) and 1000 bootstrap replications [72]. The Arabic numberals are bootstrap values, roman numerals indicate the three phylogenetic groups identified. Colinear genes and TEs are connected by shaded areas. **B**. Mapping of sRNA and mRNA libraries against one BAC from each phylogenetic group (I, II and III) and SHCRBa_035_B09 and SHCRBa_196_O13. Both sRNA and mRNA mappings are to scale. Dotted ovals indicate sRNA and mRNA peaks discussed in the text. Y axis show the mRNA and sRNA mapping density, that correspond to the proportion of mRNA or sRNA mapping in each base, normalized by the BAC size.

The largest group of sugarcane BACs that mapped to a single region within a sorghum chromosome was analyzed to evaluate hom(e)ologous and genome size diversification. These were twelve BACs selected using a probe for the single copy *rpa1a* (replication protein A1a) gene. RPA1A is known to play an essential role in DNA repair in rice [26]. In addition to the *rpa1a* gene, the BACs contained seven additional genes and a number of incomplete and putatively full-length LTR retrotransposons and DNA transposons (Figure 5A). Structural analyses indicate that these BACs represent a number of hom(e)ologous regions. The colinearity was interrupted mainly by differential distribution and size of TE insertions. A comparison with the orthologous region of the sorghum genome (chromosome 4) confirms that the sugarcane genome has longer intergenic regions than sorghum, as described above.

The CDSs of the six genes shared by the 12 BACs and sorghum were aligned and concatenated to construct a phylogenetic tree (Figure 5A). Most of the sugarcane sequences fell into three well-supported groups (I, II and III) that were in agreement with the structural analysis. BACs SHCRBa_232_H22 and SHCRBa_227_O17 did not group with BACs that they were structurally closely related to, but fell in a separated, less related group (group III). BACs SHCRBa_035_B09 and SHCRBa_196_O13 fell outside all these groups. Interestingly, the topology of the phylogeny based on the concatenated CDSs generally reflects differences of TE content in the region examined. The main structural variation between the BACs in groups I and II is the presence of a DNA transposon between the fifth and sixth genes in group I, instead of the *Harbinger* cluster found in group II. No structural variation between groups II and III was detected, apart from a variant region downstream from the sixth gene, which contained several copies of different LTR retrotransposons.

The BACs selected using the *rpa1a* gene were also mapped against the SUCEST database (EST sequences) and the RNA-seq and sRNA libraries. Figure 5B shows mapping for one BAC of each of the three phylogenetic groups (I, II and III) and the other two BACs (SHCRBa_035_B09 and SHCRBa_196_O13). While most of the mRNA transcripts mapped against the CDSs, some sRNAs also mapped against TE sequences or non-coding regions, as expected. Two expression patterns were of particular note. First, there was a common region in all BACs, downstream from a LTR retrotransposon, with peaks of mRNA sequences (Figure 5B*i*). Transcription of this region may be directed by promoter sequences in the 3′ LTR of the LTR retrotransposon. Alternatively, there may be an unidentified gene in this region. Second, differential hom(e)ologs expression was identified (Figure 5B*ii*). There are different sRNA patterns between BACs in the region between the *rpa1a* gene (white arrow) and gene 5. No sRNA reads were mapped to BACs SHCRBa_035_B09 or SHCRBa_196_O13, in which the intergenic region between the *rpa1a* gene and gene 5 is the shortest (1030 and 866 bp). On the other hand, in BACs SHCRBa_101_B12, SHCRBa_201_D09 and SHCRBa_232_h22, where the intergenic regions are longer (1556, 1556 and 1553 bp, respectively), there is also a higher number of mapped sRNAs. The main difference in the intergenic region in the BAC with the lowest (SHCRBa_196_O13) and the highest (SHCRBa_101_B12) sRNAs number of reads is the presence of DNA transposon fragments and a *Harbinger* TE in BAC SHCRBa_101_B12 (not shown in diagram), which support current models that these elements contribute to gene modulation [27].

## Discussion

The present study releases the largest and most diverse collection of sugarcane genomic regions to date. Based on comparative analysis, these regions are distributed throughout all sorghum chromosomes and are chiefly euchromatic. An understanding of these genomic regions will increase our knowledge of the structure of the sugarcane genome. The selected BAC collection includes genes known to be expressed and reveals a diverse set of sugarcane sequences associated with major biological processes. Insight into transcriptional patterns and epigenetic regulation were provided by the complementary RNA sequencing approaches.

Previous studies have shown that there is a high level of colinearity, gene structure, and sequence conservation between sorghum and sugarcane [28–31]. However, these reports conflict in terms of whether the sugarcane genome is expanding, or has expanded, relative to the sorghum genome or vice versa. Our data, based on a much larger sampling of linear genomic sequence, and assembled regions (about 100,000 bases per BAC), confirms the colinearity and conservation between the sorghum and sugarcane genomes. It also suggests that overall the sugarcane genome has undergone or is undergoing expansion within euchromatic regions compared with sorghum. This expansion is highly variable depending on the syntenic block examined (Additional file 13: Table S6), possibly explaining why the previous reports are conflicting. Nearly one-fourth of the sugarcane genome expansion compared with the sorghum can be attributed to differences in TE content, largely LTR retrotransposons. The presence of these dynamic elements within euchromatic regions may act as key factors in chromosome rearrangements, gene gain and loss, as well as epigenetic marks. Similar mechanisms have been shown to be associated with TEs in other grass genomes such as maize [32–34].

BACs from repetitive genomic regions were examined. Among these was a BAC composed entirely of *45S* ribosomal units. A consistent variation in signal intensity from chromosome to chromosome was observed using rDNA and pericentromeric and/or centromeric BACs as probes for FISH (Additional file 12: Figure S7C). Centromeres within a species are generally composed of the same types of repeats, while the abundance and arrangement of repeats can vary both between and within species [35]. Given the hybrid nature of sugarcane modern cultivars, variation in the pericentromeric/centromeric BAC signal may therefore be a reflection of the differences in pericentromeric/centromeric composition within or between the parental species. Based on previous findings using the same cultivar, the less intense interstitial rDNA signals are on *S. spontaneum* chromosomes, while the more intense terminal signals are on *S. officinarum* chromosomes [36]. While the rDNA genes are highly conserved, ITS sequence divergence can be used to resolve species relationships within a genus. Following polyploidization events, ITS units can suffer several different fates, depending on the species and time since polyploidization, for example, loss of one parental type, or homogenization [37]. It would appear that the position of the rDNA units from both parental species have been retained in modern sugarcane cultivars.

We estimate that almost one-half of the sugarcane BAC sequences are TEs. This estimate is close to that based on BAC-end sequences (BESs) from two sugarcane cultivars, R570 (42.8%) [24] and SP80-3280 (45.16%) [31]. In general, as genome size increases the proportion of the genome composed of repeats increases [38]. A significant proportion of grass genomes are composed of repetitive sequences, 40%, 62% and 82% for rice (420 Mb), sorghum (740 Mb), and maize (2160 Mb), respectively [25, 32, 39]. The basic genome size (1 ×) for *S. officinarum*, the main component of modern sugarcane cultivars genome, is 930 Mb [6], larger than sorghum (740 Mb). *S. spontaneum,* also one of the ancestors of modern sugarcane cultivars, is 750 Mb [6], similar to sorghum. The total monoploid genome size of the R570 modern cultivar, however, is 1 Gb. The percent of the sugarcane genome composed of repeats based on the BAC sequences is most likely an underestimate because the BACs were mainly from euchromatic gene-rich regions. Nevertheless, the low percent of repeats compared to genomes of a comparable size may also be a reflection of the size of modern sugarcane cultivar genome as a result of polyploidization events rather than as the result of massive TE expansion.

We examined all hom(e)ologous regions identified containing the *rpa1a* gene to better understand the consequences of polyploidization in terms of genome structure and regulation. Most of the structural variation among the BACs was due to variability in TE insertion patterns, although the topology of the phylogenetic tree inferred using the coding gene sequences reflects structural variation between hom(e)ologous regions. The topology indicates that there is at least three well-defined haplogroups in this region. We speculate that these haplotypes are derived from the parental species. The 10 BACs from the groups A, B, and C (approximately 80% of the BACs) were inherited from *S. officinarum* and the two remaining from *S. spontaneum*. We were not able to evaluate if there is any selective constraint driving the diversification of the putative hom(e)ologous sequences from *S. officinarum* as haplogroups. Further sequencing of this region in *S. officinarum* and *S. spontaneum* may identify any selective constraint.

The high conservation of gene content and colinearity between sugarcane haplotypes has been previously shown, here we confirm this finding analyzing more hom(e)ologs of a single region [29, 30]. These results contrast with the high DNA sequence elimination and recombination observed between hom(e)ologous chromosomes in allopolyploid wheat and other monocot and eudicot plants (see Liu et al. [40] for a review). In all these cases, it is not clear if the changes occurred immediately after polyploidization. We consider that the high conservation is not due to the gene richness of the regions, since the gene frequency in the *rpa1a* BACs is much lower than those regions studied in previous colinearity studies in sugarcane [28–30]. There are several possible reasons for the low recombination rate in this region. The hybridization events resulting in the modern sugarcane cultivar are very recent, within the last two centuries, and it has been estimated that there have been few meiotic divisions since [3]. The parental species themselves have recently diverged, between 1.5 and 2.0 million years ago. Finally, the evolution of the haplogroups in this polyploid genome may have been shaped by the phenomenon of pairing behavior, which favors the transmission of non-mutated chromosomes to the progeny [30].

We mapped sRNA and mRNA libraries against the *rpa1a* region. The results show that the hom(e)ologous BACs have differential mapping patterns for both kinds of RNAs. Most of the variation was observed in promoters, TEs and intergenic sequences. The promoter regions within the LTRs at each end of an LTR retrotransposon can act as novel promoters or enhancers, driving changes in host-gene expression patterns [41]. There are peaks of RNA (ESTs and RNA-seq) mapping in a region downstream to an LTR retrotransposon, where no non-TE-coding genes have been identified. Promoters within the 3′ LTR region may be driving expression of this region in an allelic dependent manner. The region between two host genes is variable both in length and for the presence/absence of TE fragments. Peaks of sRNA mapped between the two non-TE coding genes correlates with the intergenic length and the presence of TEs. Several studies have shown that hom(e)ologous diversity needs to be evaluated not only in terms of gene coding DNA, but also in terms of regulatory regions, since regulatory regions have important roles in genetic control and are under independent evolutionary pressures [42]. This can be particularly important in polyploids, due to the high number of hom(e)ologous loci. In the *rpa1a* BACs there are indications of mRNA and sRNA variation, as evidenced by the sRNA and transcriptome mapping, that could influence gene expression and function. Interestingly, the two most highly expressed *rpa1a* hom(e)ologs correspond to a BAC that did not cluster within the three main haplotypes and another from cluster III.

Crop genomics is being used to increase the effectiveness of breeding, since traits of interest can be selected more precisely, directly and cost-effectively [43]. For over 10 years directed genetic modification of sugarcane has been a reality in laboratories with field trials also being conducted [44]. Genomics could also aid in traditional marker-based breeding, providing putative marker sequences derived from genes, TEs, intergenic or low complexity regions [45–47]. Here we have sequenced and annotated 1,400 sugarcane protein-coding genes and several non-coding genes, including ribosomal and miRNA genes. The protein-coding genes code for enzymes in several metabolic pathways. For some of the genes that are clearly important in sugarcane breeding, for example, genes from the sucrose and starch metabolism, the complete CDSs are available in the transcriptome database, but no information about introns and intergenic regions have been previously published. Other genes have not been previously sequenced in sugarcane, among them genes involved in the metabolic pathways not traditionally considered in sugarcane breeding, such as the carotenoid biosynthesis pathway. The sequencing of complete genes, including coding regions, UTRs, introns and promoters in genomic sequencing projects, instead of sequencing only transcribed sequences, as in transcriptome sequencing projects, provides a broader database for the design of transgene constructs. This work has shown that it is fundamental to combine genome and transcriptome sequencing approaches (sRNA and mRNA) to validate genome annotation and provide a broad understanding of functional genomics.

The potential of the sequenced BAC collection was demonstrated by sequencing 16 genes related to the central enzymatic steps of carbon partitioning in source-sink-growth in plants. Three main conclusions were drawn. First, the sequenced regions enabled the identification of differential expression levels in specific enzymatic steps in actively growing bud tissue. Second, we were able to differentiate the expression of paralogous loci. Finally, a previously unreported gene duplication was described for the *s6pp* gene in sugarcane and sorghum. Examination of a region covering the intergenic region and part of the two genes from a commercial hybrid breeding panel, *S. officinarum*, *S. spontaneum*, *Miscanthus* sp and the sorghum genome shows that *S. spontaneum* did not contribute to the haplotype identified in hybrid cultivars. Interestingly, the *Miscanthus* sp. sequences fell into four major haplotype groups. Another haplotype group consisted exclusively of commercial hybrids. Sequence variability among paralogous or hom(e)ologous allelic loci has to be considered, since the most effective gene copy should be selected in order to avoid non-additive effects [42]. Thus, it is essential to sequence further candidate hom(e)ologous regions that have the potential to be valuable in transgenic breeding, in order to increase our knowledge of sugarcane gene variability.

## Conclusion

The genome sequence released in the present work contributes towards a fundamental understanding of the structure of the sugarcane genome. The present data will also contribute to improving our understanding of the genetic basis of sucrose content and physiology, providing molecular tools for breeding purposes and gene discovery related to traits such as plant defense, metabolism, flowering, and responses to biotic and abiotic stresses.

## Methods

### Sugarcane BAC library and BAC selection

Two approaches were used to select BACs from the sugarcane hybrid R570 [7]: macro-array hybridization using PCR-amplified and overgo probes, and 3D pool screening by real-time PCR. Overgo probes were designed using the BACMAN database [48] and were used as queries in a BLASTn search against the sorghum (v1.0) [14] and rice (v6.1) [49] genome assemblies. Since the overgo probes are only 40 nts in length, we used a series of cut-off values; for matches from 40 to 38 nt (allowing for 2 mismatches for 40 nt or 1 mismatch for 39 nt and no mismatches for 38 nt alignments). Only those probes that hybridized to 1–10 BACs were used to select BACs for sequencing to reduce false positives and to exclude multigene families.

Macro-array hybridizations were performed according the manufacturer’s instruction and Bowers et al. [50]. After purification of the PCR fragment using the GFX PCR DNA and Gel Band Purification Kit (GE Healthcare) the probes were labeled using the Random Primer DNA Labeling System (Invitrogen), following the manufacturer’s instructions. 3D pools were constructed according to Adam-Blondon et al. [51]. Briefly, the 269 plates of the SHCRBa library were arranged in 11 blocks of 24 plates and the BACs pooled by plate, line and row in growth medium. Each pool was amplified using the Phi29 enzyme from the IllustraGenomi Phi V2 DNA Amplification Kit (GE Healthcare), according the manufacturer’s instructions. We screened for specific markers by RT-PCR as follows: in a final volume of 5 μl, 100 ng of the pool DNA was amplified using 0.4 μM of each primer and 1 X SYBR Green Master Mix (Roche). The cycling parameters used for amplification were 95°C for 5 min for initial denaturation, 40 cycles of 95°C for 20 sec, 60°C for 20 sec and 72°C for 40 sec.

### BAC sequencing and assembling

Three hundred and thirteen BACs were sequenced using the 454/Roche sequencing platform and four were sequenced using Sanger/ABI technology. BAC DNA was extracted using the QIAGEN Large-Construct Kit, following the manufacturer’s protocols. 454 DNA libraries were prepared using either the General or the RAPID GS FLX Titanium Library Preparation Kit, individually emPCR amplified and sequenced using 454 Titanium kits, according the manufacturer’s specifications and default parameters. Nine different gasket pooling strategies were tested (Additional file 2: Table S1). Sanger sequencing was performed according Manetti et al. [52]. The sequencing reads were assembled using Phrap [53] with different parameter values, depending on the results of the first assembly, which was performed using default parameters and repeat_stringency set at 0.3 (for ease of contig joining). Given the deep coverage achieved for each BAC, all sequences were used for assembly. Bad quality reads were automatically kept as singletons by Phrap [53]. Medium-to-low quality reads were used in the assembly because, given the high coverage, the impact of these medium-to-low quality reads is expected to be low. This strategy was adopted because in regions that are difficult to sequence (e.g., homopolimeric regions), median-to-low quality reads could help to close gaps or at least confirm a scaffold.

### Gene and repeat annotation

BACs were first annotated using an automated pipeline for identification of genes and TEs based on *de novo* prediction and BLASTx. TEs were screened using RepeatMasker [54] against repeats from Viridiplantae [55] and sugarcane LTR retrotransposons [10], with a cut-off score of 250. Both complete and incomplete elements were identified. MITE Hunter [56] with default parameters was used to extract all MITEs from the BACs. The alignment files generated were grouped together using clans [57] and screened using RepeatMasker [54]. MITEs were then classified according to type of target site duplication and terminal inverted repeat features [58].

Genes were annotated using masked BACs, using the software programs Augustus [59], Glimmer HMM [60], PASA [61], Evidence Modeler [62], SignalP [63], and TMHMM [64]. Exon-intron boundaries were examined using Artemis [65], compared to results based on BLAST alignments against sugarcane ESTs [8] and annotated sorghum and rice proteins [14, 66, 67] and adjusted if necessary. If no hits against sugarcane ESTs, rice and sorghum protein sequences were found, the predicted ORF was not modified. Validation of splice sites were performed by GenBank tools at sequence submission. Lastly, putative intergenic sequences were BLASTx (nr database) screened for additional genes not annotated by the *de novo* prediction programs. Manual categorization was performed according to the SUCEST project database [8]. Blast2GO analyses were performed as previously described [68] using BLASTp with an e-value cut-off of e^-10^. Screening for mitochondrial and chloroplast sequence contamination was done by a BLASTn of the sugarcane organellar genomes (GenBank: NC_005878 and NC_008360).

We used the CDSs from the BACs, the 43,141 SASs [69] and the KeggMapper tool available at MG-RAST [70] for global automatic mapping with cut-offs of an e-value of e^-5^, 60% identity, and a minimum alignment length of 15 bp. We used the sugarcane CDSs and the KEGG Automatic Annotation Server [71], with the BBH search option, to map sucrose and starch pathways genes.

All BAC sequences generated in this study, with protein-coding gene and full-length TE annotation, can be accessed in the GenBank database under accession numbers [GenBank: KF184657 to KF184973].

### Colinearity and synteny analyses

Two analyses were performed to confirm that the BACs represent a homogeneous sampling of the sugarcane genome, and to evaluate colinearity and synteny with the sorghum genome. First, we estimated the chromosomal location of the BACs in the sorghum genome by a BLASTn analysis, using sugarcane non-TE coding sequences as queries against the sorghum genome (v1.0) [25]. We then checked this localization by blasting the predicted protein sequences against the masked sorghum protein database [14]. The chromosomal location of the BAC in the sorghum genome was directly assigned for predicted protein sequences with single-hits. A high-throughput maximum-likelihood phylogenetic analysis [23] was applied for CDSs with multiple-hits using the sorghum predicted proteome [14]. Redundant sugarcane predicted protein sequences from putative hom(e)ologous BACs were manually evaluated. Sorghum orthologous genes assigned to more than one BAC were examined when there were two or more colinear predicted protein sequences on a single BAC. Redundant predicted protein sequences were removed from the analysis. A Welch’s t test was applied to check the size variation hypothesis.

The structure of the 12 BACs selected using the *rpa1a* gene as probe was analyzed in detail using Artemis [65]. The CDSs of the six genes shared by the 12 BACs and sorghum were aligned and concatenated. A neighbor-joining phylogenetic tree was inferred with using the highest ranked substitution model (Tajima-Nei) and 1000 bootstrap replications, using MEGA 5 [72].

### Analysis of ribosomal and centromeric BACs

The BAC SCHRBa_039_D18 was initially identified through the gene annotation as containing ribosomal genes. The region syntenic to the BAC in rice, maize, sorghum and *Arabidopsis* was identified using CoGE [73]. The top 2 hits from BLASTn where the description was not an unidentified sequence were downloaded and manually aligned using BioEdit [74] with BAC SCHRBa_039_D18 to determine the beginning and end of each ribosomal gene.

Three BACs were identified as putatively pericentromeric and/or centromeric by the absence of coding genes during the repeat annotation. The nucleotide sequence of the BACs were further analyzed by appropriate BLASTs against the SCEN repeat [21]. LTR nucleotide sequence and conceptually translated coding domains from the sugarcane curated LTR retrotransposon database [10]. The centromeric repeat, SCEN, was extracted and aligned using ClustalW in BioEdit [74], and the pairwise % identity calculated using Genious [75].

### Fluorescence in situ hybridization (FISH)

Distribution of the sugarcane ribosomal (SCHRBa_039_D18) BAC and a pericentromeric/centromeric (SCHRBa_239_N21) BAC were analyzed by FISH on metaphase chromosomes. FISH procedures were as described in [10], except for preparation of the probes and blocking DNA. All kits were used according to the manufacturer’s instructions. One μg of each BAC was used in a 20 μL nick translation reaction using the NT mix (Roche) with Digoxigenin (DIG)-11-dUTP (Invitrogen) or Biotin-16-dUTP (Invitrogen). Labeling efficiency was tested according to Heslop-Harrison and Schwarzacher [76] (protocol 4.7). Blocking DNA was prepared from genomic DNA from the sugarcane cultivar SP80-3280. Genomic DNA was extracted from meristem according to Aljanabi et al. [77], except that the meristem was first ground in liquid nitrogen before adding the homogenization buffer. The genomic DNA was sheared by placing it at 95°C until it was less than 1 Kb in size.

### sRNA analysis

Raw sequences [10] were retrieved in a FASTQ formatted file and the adapter sequences were removed using Perl Scripts. Reads in the size 20–25 nucleotides were sorted into two separate files, 20–22 nt and 23–25 nt for subsequent analyses. We used the MAQ software [78] to map the collection of sRNA reads against the BACs. We used the low stringent cut-off parameter of 0–2 nt mismatches because the BACs and sRNA reads were derived from different sugarcane cultivars. Graphical representations of sRNA mapping was created using the SeqMonk software [79].

### RNA-seq sequencing and analysis

A sugarcane transcriptome was constructed from germinating shoot axillary buds five days after planting. Single budded setts from the sugarcane variety RB92-5345 were placed in trays with buds facing upwards, covered with moist vermiculite, and incubated at 26–30°C under greenhouse conditions. Total RNA was extracted from pooled breaking buds using a lithium chloride protocol [80]. For the construction of RNA-seq libraries, all procedures were carried out according to Illumina’s instructions using the ‘TruSeq RNA Sample Prep v2 Low Throughput (LT)’ kit. The libraries were paired-end sequenced on the Illumina system (HiScanSQ) (GA3 – ESALQ-USP). Sequencing reads were mapped using the Burrows-Wheeler Aligner BWA [81] and the SAM tools [82]. The RNA-seq library can be accessed in the NCBI high-throughput DNA and RNA sequence read archive under the accession number [SRA: SRX500284].

### *S6pp*tandem gene duplication network analysis

The evolutionary relationship of the putative tandem gene duplication of the *s6pp* loci in two clones *S. spontaneum* (Mandalay and IN8458), one *S. officinarium* (Badila), and 11 modern sugarcane hybrid cultivars (R570, SP80-3280, SP70-1143, RB835486, RB72454, RB867515, Co-290, POJ2878, NCo-310, NA5679 and SP81-3250), one *Miscanthus* species, and the sorghum genome was evaluated by sequencing and network analysis. The 1539 bp region was first identified in the SHCRBa_104_G22 BAC (position 2977 to 4515, Additional file 14: Figure S8). Sequence for the sorghum genome was taken from published sequence (3,984,822 to 3,988,630 nt in chromosome_9). The primers 2995 F and 4500R, were used to amplify the fragment from the other cultivars and species (Additional file 14: Figure S8). PCR reactions were performed in a final volume of 25 μL, using 50 ng of genomic DNA, 0.4 μM of each primer, 0.2 mM of each dNTP, and 0.5 μL Elongase Enzyme Mix (Invitrogen) in 0.5 X PCR buffer A and 0.5 X PCR buffer B. The cycling parameters used for amplification were: 94°C for 10 min for initial denaturation, 35 cycles of 94°C for 30 sec, 55°C for 30 sec, and 68°C for 6 min. The fragments obtained were purified directly from the PCR product, using the NucleoSpin Extract II (Macherey-Nagel), and cloned into the pGEM-T EasyVector System (Promega). Seven to 15 randomly chosen clones from each sample were automatically sequenced in an ABI PRISM 3730 (Applied Biosystems) using the primers M13F and M13R, and the six internal primers (Additional file 14: Figure S8). The following PCR conditions were used: in a final volume of 10 μL, 300 ng of plasmid DNA, 1 μM of each primer, 2 μL of BigDye Terminator v3.1 (Applied Biosystems) in 1 X BigDye buffer. Sequence alignment was performed using Clustal W [83] and the reduced median-joining network analysis using the NETWORK 4.5.1.0 software with default parameters [84]. The sequences and alignments are available on request.

### Availability of supporting data

The BAC sequence data set supporting the results of this article is available in the GenBank repository [KF184657 to KF184973 at http://www.ncbi.nlm.nih.gov/genbank], in the CoGe website [Saccharum hybrid cultivar R570 (id23984) in https://genomevolution.org/CoGe/] and in the GaTElab website, as a GBrowser search tool [ https://gate.ib.usp.br/GateWeb/en/gbrowse-pagina]. The RNA-seq library data set is available in the Sequence Read Archive (SRA) repository [SRX500284 in http://www.ncbi.nlm.nih.gov/sra].

## Electronic supplementary material

Additional file 1: Figure S1: Schematic diagram of evolutionary history of grasses and sugarcane. BEP clade: Bambusoideae, Ehrhartoideae and Pooideae subfamilies. Numbers indicate divergence times [85]. (PDF 1 MB)

Additional file 2: Table S1: Summary of BAC selection and sequencing data. For each BAC, the selection method and marker used to select the BAC, the number of reads, mean read length, mean sequencing quality, coverage, number of contigs and the length of the BAC is shown. *: BACs sequenced using Sanger method; na: not applicable. (XLSX 52 KB)

Additional file 3: Table S2: Transposable element annotation for each BAC. Annotation was performed using RepeatMasker [54] against the Viridiplantae Repbase database [9] and sugarcane LTR retrotransposons [10]. (XLSX 171 KB)

Additional file 4: Table S3: Summary of repeat content of sugarcane BACs. (PDF 184 KB)

Additional file 5: Figure S2: Distribution of Blast2GO annotations of protein-coding sequences. The chart shows level 2 annotations for A) Biological Processes, B) Molecular Function and C) Cellular Components. (PDF 144 KB)

Additional file 6: Figure S3: BLASTp best match distribution by species of the sugarcane putative protein-coding gene collection against the NCBI nr database. The species with the highest number of top-hits is *S. bicolor*, with 908 matches. (PDF 236 KB)

Additional file 7: Figure S4: Annotation of sugarcane predicted protein-coding genes according to the 17 functional categories used in the sugarcane transcriptome study. The classification was done using the BLAST2GO tool (e-value < e^-10^). The 18^th^ category “mobile genetic elements” proposed by Vettore et al. [8] was not included in this analysis since TE-derived genes were not included in the gene annotation. (PDF 502 KB)

Additional file 8: Figure S5: KeggMapper plot showing global metabolic pathways. Red lines indicate reactions for which predicted enzyme-coding genes were identified by sugarcane SASs, blue line indicate those identified by CDSs from sugarcane BACs and pink lines indicate those identified by both SASs and CDSs. Note that a single line may represent more than one match. (PDF 2 MB)

Additional file 9: Table S4: Detailed results for the KeggMapper study. EC numbers are given for predicted enzyme coding genes identified from SASs, CDS in BACs and from both SASs and CDS. (XLSX 29 KB)

Additional file 10: Table S5: Detailed annotation of the sucrose and starch pathway genes and RNA-seq mapping. (PDF 187 KB)

Additional file 11: Figure S6: Global overview of sRNA mapping along sugarcane BACs. The horizontal colored bars shows extent of coverage. The colours of the bars are scaled from low (dark blue) to medium (green) to high (red). The rDNA BAC (SHCRBa_039_D18) has the highest number of sRNAs mapped. (PDF 4 MB)

Additional file 12: Figure S7: Structural organization and chromosomal location of the rDNA and the pericentromeric and/or centromeric BACs. A. Structure of the *45S* ribosomal transcription unit identified in BAC (SCHRBa_039_D18). The BAC consists of 14 copies of the unit, one in reverse orientation to the other 13. ITS = internal transcribed spacer, ETS = external transcribed spacer, IGS = intergenic spacer. B. Simplified schematic of the centromeric BAC (SCHRBa_239_N21). The LTRs of the LTR retrotranspons are shown as arrows, the internal domains as squares. Each LTR retrotransposon is numbered consecutively. Black arrows indicate the location of the insertion of one element into another. ‘COPIA’ is an unidentified *Copia-*like element. C. Localization of the ribosomal (SCHRBa_039_D18) and pericentromeric/centromeric (SCHRBa_239_N21) BACs to metaphases from root tips of the sugarcane cultivar R570. Metaphases are counterstained with DAPI (blue). The centromeric BAC was detected with anti-digoxigenin-rhodamine (red), the ribosomal BAC with NeutrAvidin-Oregon Green-488 (green). (PDF 451 KB)

Additional file 13: Table S6: Detailed colinearity study between sugarcane BACs with sorghum genome. Alternate white and gray blocks are used to visually separate BACs and double-lines indicate break of perfect colinearity. (XLSX 76 KB)

Additional file 14: Figure S8: Location of the primers used to amplify the *s6pp* tandem gene duplication region. A 1539 bp fragment spanning the region was amplified from *S. spontaneum*, S. *officinarum*, modern sugarcane hybrid cultivars, *Miscanthus* sp. and sorghum using the primer pair 2995F and 4500R. All other primers are internal sequencing primers. Black bars indicate the two *s6pp* genes. 2995F: 5′ GCA GGG AGC GAG CAC ACG TT 3′, 4500R: 5′ TCG GTG CTC TCC CCT GCG AA 3′, 3223F: 5′ ACG ACC TTG CCT CTC TGT TG 3′, 4231R: 5′ TCA ACT TGT GAG GGA GAG CA 3′, 3404F: 5′ CAA TCG CTG TCG ATG GTG GC 3′, 4144R: 5′ CTG GCT GTA TCC GTA CAG AGG 3′, 3618R: 5′ AAG CTC TTG CCA GGA TTG CT 3′ and 3811F: 5′ GGC CGA GTT CTC CCA TGA TT 3′. (PDF 1 MB)

## Abbreviations

CDS: Coding DNA sequence
CRM: Centromeric-specific retrotransposon of maize
GO: Gene Ontology
IGS: InterGenic spacer
ITS: Internal transcribed spacer
miRNA: microRNA
MITE: Miniature inverted-repeat transposable element
pri-miRNA: Primary miRNA
rasiRNA: Repeated-associated small interfering RNA
rDNA: Ribosomal DNA
S6PP: Sucrose-6-Phosphate Phosphohydrolase
SAS: Sugarcane assembled EST sequence
siRNA: Small interfering RNA
SSR: Single sequence repeats
SUCEST: Sugarcane EST project
TE: Transposable element.

## References

[1] **European Commission: Agriculture and Rural Development: Sugar** http://ec.europa.eu/agriculture/sugar/index_en.htm

[2] Kellogg EA (2001). Evolutionary history of the grasses. Plant Physiol.

[3] Grivet L, Arruda P (2001). Sugarcane genomics: depicting the complex genome of an important tropical crop. Curr Opin Plant Biol.

[4] Piperidis G, Piperidis N, D’Hont A (2010). Molecular cytogenetic investigation of chromosome composition and transmission in sugarcane. Mol Genet Genomics.

[5] D’Hont A (2005). Unraveling the genome structure of polyploids using FISH and GISH; examples of sugarcane and banana. Cytogenet Genome Res.

[6] D’Hont A, Glaszmann JC (2001). Sugarcane genome analysis with molecular markers: a first decade of research. Int Soc Sugar Cane Technol Proc XXIV Congr.

[7] Tomkins J, Yu Y, Miller-Smith H, Frisch D, Woo S, Wing R (1999). A bacterial artificial chromosome library for sugarcane. Theor Appl Genet.

[8] Vettore L, Silva FR, Kemper EL, Souza GM, Silva AM, Ferro M, Henrique-Silva F, Giglioti ÉA, Lemos MVF, Coutinho LL, Nobrega MP, Carrer H, França SC, Bacci MJ, Goldman MHS, Gomes SL, Nunes LR, Camargo LEA, Siqueira WJ, Van Sluys M-A, Thiemann OH, Kuramae EE, Santelli RV, Marino CL, Targon MLPN, Ferro JA, Silveira HCS, Marini DC, Lemos EGM, Monteiro-Vitorello CB (2003). Analysis and functional annotation of an expressed sequence tag collection for tropical crop sugarcane. Genome Res.

[9] **Repbase** http://www.girinst.org/repbase/

[10] Domingues DS, Cruz GMQ, Metcalfe CJ, Nogueira FTS, Vicentini R, Alves C de S, Van Sluys M-A (2012). Analysis of plant LTR-retrotransposons at the fine-scale family level reveals individual molecular patterns. BMC Genomics.

[11] **National Center for Biotechnology Information (NCBI)** http://www.ncbi.nlm.nih.gov/

[12] Meyer F, Paarmann D, D’Souza M, Olson R, Glass EM, Kubal M, Paczian T, Rodriguez A, Stevens R, Wilke A, Wilkening J, Edwards RA (2008). The metagenomics RAST server - a public resource for the automatic phylogenetic and functional analysis of metagenomes. BMC Bioinformatics.

[13] Keeling PL, Myers AM (2010). Biochemistry and genetics of starch synthesis. Annu Rev Food Sci Technol.

[14] **Phytozome v9.1: Home** http://www.phytozome.net/

[15] Dias ES, Carareto CMA (2012). Ancestral polymorphism and recent invasion of transposable elements in Drosophila species. BMC Evol Biol.

[16] Posada D, Crandall K (2001). Intraspecific gene genealogies: trees grafting into networks. Trends Ecol Evol.

[17] Swaminathan K, Alabady MS, Varala K, De Paoli E, Ho I, Rokhsar DS, Arumuganathan AK, Ming R, Green PJ, Meyers BC, Moose SP, Hudson ME (2010). Genomic and small RNA sequencing of Miscanthus x giganteus shows the utility of sorghum as a reference genome sequence for Andropogoneae grasses. Genome Biol.

[18] Zanca AS, Vicentini R, Ortiz-Morea FA, Del Bem LE, da Silva MJ, Vincentz M, Nogueira FT (2010). Identification and expression analysis of microRNAs and targets in the biofuel crop sugarcane. BMC Plant Biol.

[19] Piriyapongsa J, Jordan IK (2007). A family of human microRNA genes from miniature inverted-repeat transposable elements. PLoS ONE.

[20] Barrera-Figueroa BE, Gao L, Wu Z, Zhou X, Zhu J, Jin H, Liu R, Zhu J-K (2012). High throughput sequencing reveals novel and abiotic stress-regulated microRNAs in the inflorescences of rice. BMC Plant Biol.

[21] Nagaki K, Tsujimoto H, Sasakuma T (1998). A novel repetitive sequence of sugar cane, SCEN family, locating on centromeric regions. Chromosom Res.

[22] Nagaki K, Neumann P, Zhang D, Ouyang S, Buell CR, Cheng Z, Jiang J (2005). Structure, divergence, and distribution of the CRR centromeric retrotransposon family in rice. Mol Biol Evol.

[23] Vicentini R, Del Bem LE, Van Sluys M-A, Nogueira F, Vincentz M (2012). Gene content analysis of sugarcane public ESTs reveals thousands of missing coding-genes and an unexpected pool of grasses conserved ncRNAs. Trop Plant Biol.

[24] Kim C, Lee T-H, Compton RO, Robertson JS, Pierce GJ, Paterson AH (2013). A genome-wide BAC end-sequence survey of sugarcane elucidates genome composition, and identifies BACs covering much of the euchromatin. Plant Mol Biol.

[25] Paterson AH, Bowers JE, Bruggmann R, Dubchak I, Grimwood J, Gundlach H, Haberer G, Hellsten U, Mitros T, Poliakov A, Schmutz J, Spannagl M, Tang H, Wang X, Wicker T, Bharti AK, Chapman J, Feltus FA, Gowik U, Grigoriev IV, Lyons E, Maher CA, Martis M, Narechania A, Otillar RP, Penning BW, Salamov AA, Wang Y, Zhang L, Carpita NC (2009). The Sorghum bicolor genome and the diversification of grasses. Nature.

[26] Chang Y, Gong L, Yuan W, Li X, Chen G, Li X, Zhang Q, Wu C (2009). Replication protein A (RPA1a) is required for meiotic and somatic DNA repair but is dispensable for DNA replication and homologous recombination in rice. Plant Physiol.

[27] Feschotte C (2008). Transposable elements and the evolution of regulatory networks. Nat Rev Genet.

[28] Wang J, Roe B, Macmil S, Yu Q, Murray JE, Tang H, Chen C, Najar F, Wiley G, Bowers J, Van Sluys M-A, Rokhsar DS, Hudson ME, Moose SP, Paterson AH, Ming R (2010). Microcollinearity between autopolyploid sugarcane and diploid sorghum genomes. BMC Genomics.

[29] Garsmeur O, Charron C, Bocs S, Jouffe V, Samain S, Couloux A, Droc G, Zini C, Glaszmann J-C, Van Sluys M-A, D’Hont A (2011). High homologous gene conservation despite extreme autopolyploid redundancy in sugarcane. New Phytol.

[30] Jannoo N, Grivet L, Chantret N, Garsmeur O, Glaszmann JC, Arruda P, D’Hont A (2007). Orthologous comparison in a gene-rich region among grasses reveals stability in the sugarcane polyploid genome. Plant J.

[31] Figueira TRES, Okura V, da Silva FR, da Silva MJ, Kudrna D, Ammiraju JSS, Talag J, Wing R, Arruda P (2012). A BAC library of the SP80–3280 sugarcane variety (saccharum sp.) and its inferred microsynteny with the sorghum genome. BMC Res Notes.

[32] Schnable PS, Ware D, Fulton RS, Stein JC, Wei F, Pasternak S, Liang C, Zhang J, Fulton L, Graves TA, Minx P, Reily AD, Courtney L, Kruchowski SS, Tomlinson C, Strong C, Delehaunty K, Fronick C, Courtney B, Rock SM, Belter E, Du F, Kim K, Abbott RM, Cotton M, Levy A, Marchetto P, Ochoa K, Jackson SM, Gillam B (2009). The B73 maize genome: complexity, diversity, and dynamics. Science.

[33] Tenaillon MI, Hufford MB, Gaut BS, Ross-Ibarra J (2011). Genome size and transposable element content as determined by high-throughput sequencing in maize and Zea luxurians. Genome Biol Evol.

[34] Zhang J, Yu C, Krishnaswamy L, Peterson T, Birchler JA (2011). Transposable Elements as Catalysts for Chromosome Rearrangements. Methods Mol Biol.

[35] Ma J, Wing RA, Bennetzen JL, Jackson SA (2007). Plant centromere organization: a dynamic structure with conserved functions. Trends Genet.

[36] D’Hont A, Grivet L, Feldmann P, Rao S, Berding N, Glaszmann JC (1996). Characterisation of the double genome structure of modern sugarcane cultivars (*Saccharum* spp.) by molecular cytogenetics. Mol Gen Genet.

[37] Bao Y, Wendel JF, Ge S (2010). Multiple patterns of rDNA evolution following polyploidy in *Oryza*. Mol Phylogenet Evol.

[38] Lynch M (2007). The Origins of Genome Architecture.

[39] International Rice Genome Sequencing Project (2005). The map-based sequence of the rice genome. Nature.

[40] Liu B, Xu C, Zhao N, Qi B, Kimatu JN, Pang J, Han F (2009). Rapid genomic changes in polyploid wheat and related species: implications for genome evolution and genetic improvement. J Genet Genomics.

[41] Lisch D (2012). How important are transposons for plant evolution?. Nat Rev Genet.

[42] Udall JA, Wendel JF (2006). Polyploidy and crop improvement. Crop Sci.

[43] Varshney RK, Graner A, Sorrells ME (2005). Genomics-assisted breeding for crop improvement. Trends Plant Sci.

[44] Menossi M, Silva-Filho MC, Vincentz M, Van-Sluys M-A, Souza GM (2008). Sugarcane functional genomics: gene discovery for agronomic trait development. Int J Plant Genomics.

[45] Palhares AC, Rodrigues-Morais TB, Van Sluys M-A, Domingues DS, Maccheroni W, Jordão H, Souza AP, Marconi TG, Mollinari M, Gazaffi R, Garcia AAF, Vieira MLC (2012). A novel linkage map of sugarcane with evidence for clustering of retrotransposon-based markers. BMC Genet.

[46] Andersen JR, Lübberstedt T (2003). Functional markers in plants. Trends Plant Sci.

[47] Kalendar R, Flavell AJ, Ellis THN, Sjakste T, Moisy C, Schulman A (2011). Analysis of plant diversity with retrotransposon-based molecular markers. Heredity (Edinb).

[48] **PGML BACMan On The Web: Grasses** http://www.plantgenome.uga.edu/bacman/BACManwww.php

[49] **Rice Genome Annotation Project** http://rice.plantbiology.msu.edu/

[50] Bowers JE, Arias MA, Asher R, Avise JA, Ball RT, Brewer GA, Buss RW, Chen AH, Edwards TM, Estill JC, Exum HE, Goff VH, Herrick KL, Steele CLJ, Karunakaran S, Lafayette GK, Lemke C, Marler BS, Masters SL, McMillan JM, Nelson LK, Newsome GA, Nwakanma CC, Odeh RN, Phelps CA, Rarick EA, Rogers CJ, Ryan SP, Slaughter KA, Soderlund CA (2005). Comparative physical mapping links conservation of microsynteny to chromosome structure and recombination in grasses. Proc Natl Acad Sci U S A.

[51] Adam-Blondon A-F, Bernole A, Faes G, Lamoureux D, Pateyron S, Grando MS, Caboche M, Velasco R, Chalhoub B (2005). Construction and characterization of BAC libraries from major grapevine cultivars. Theor Appl Genet.

[52] Manetti ME, Rossi M, Cruz GMQ, Saccaro NL, Nakabashi M, Altebarmakian V, Rodier-Goud M, Domingues D, D’Hont A, Van Sluys MA (2012). Mutator system derivatives isolated from sugarcane genome sequence. Trop Plant Biol.

[53] **Phrap** http://www.phrap.org/

[54] **RepeatMasker** http://www.repeatmasker.org/

[55] Jurka J, Kapitonov VV, Pavlicek A, Klonowski P, Kohany O (2005). Repbase update, a database of eukaryotic repetitive elements. Cytogenet Genome Res.

[56] Han Y, Wessler SR (2010). MITE-Hunter: a program for discovering miniature inverted-repeat transposable elements from genomic sequences. Nucleic Acids Res.

[57] Frickey T, Lupas A (2004). CLANS: a Java application for visualizing protein families based on pairwise similarity. Bioinformatics.

[58] Han Y, Qin S, Wessler SR (2013). Comparison of class 2 transposable elements at superfamily resolution reveals conserved and distinct features in cereal grass genomes. BMC Genomics.

[59] Keller O, Kollmar M, Stanke M, Waack S (2011). A novel hybrid gene prediction method employing protein multiple sequence alignments. Bioinformatics.

[60] Majoros WH, Pertea M, Salzberg SL (2004). TigrScan and GlimmerHMM: two open source *ab initio* eukaryotic gene-finders. Bioinformatics.

[61] Haas BJ, Delcher AL, Mount SM, Wortman JR, Smith RK, Hannick LI, Maiti R, Ronning CM, Rusch DB, Town CD, Salzberg SL, White O (2003). Improving the *Arabidopsis* genome annotation using maximal transcript alignment assemblies. Nucleic Acids Res.

[62] Haas BJ, Salzberg SL, Zhu W, Pertea M, Allen JE, Orvis J, White O, Buell CR, Wortman JR (2008). Automated eukaryotic gene structure annotation using EVidenceModeler and the Program to assemble spliced alignments. Genome Biol.

[63] Petersen TN, Brunak S, von Heijne G, Nielsen H (2011). SignalP 4.0: discriminating signal peptides from transmembrane regions. Nat Methods.

[64] **TMHMM Server v. 2.0** http://www.cbs.dtu.dk/services/TMHMM-2.0/

[65] Rutherford K, Parkhill J, Crook J, Horsnell T, Rice P, Rajandream MA, Barrell B (2000). Artemis: sequence visualization and annotation. Bioinformatics.

[66] **UniProt** http://www.uniprot.org/

[67] **InterPro: Protein sequence analysis and classification** http://www.ebi.ac.uk/interpro/

[68] Conesa A, Götz S (2008). Blast2GO: a comprehensive suite for functional analysis in plant genomics. Int J Plant Genomics.

[69] **SUCEST-FUN Project** http://sucest-fun.org/

[70] **MG-RAST: metagenomics analysis server** http://metagenomics.anl.gov/

[71] **KAAS - KEGG automatic annotation server** http://www.genome.jp/kegg/kaas/

[72] Tamura K, Peterson D, Peterson N, Stecher G, Nei M, Kumar S (2011). MEGA5: molecular evolutionary genetics analysis using maximum likelihood, evolutionary distance, and maximum parsimony methods. Mol Biol Evol.

[73] Lyons E, Freeling M (2008). How to usefully compare homologous plant genes and chromosomes as DNA sequences. Plant J.

[74] Hall TA (1999). BioEdit: a user-friendly biological sequence alignment editor and analysis program for Windows 95/98/NT. Nucleic Acids Symp Ser.

[75] **Geneious - Homepage** http://www.geneious.com/

[76] Heslop-Harrison P, Schwarzacher T (2000). Practical In Situ Hybridization.

[77] Aljanabi S, Forget L, Dookun A (1999). An improved and rapid protocol for the isolation of polysaccharide-and polyphenol-free sugarcane DNA. Plant Mol Biol Report.

[78] **Maq: Mapping and assembly with qualities** http://maq.sourceforge.net/

[79] **SeqMonk** http://www.bioinformatics.babraham.ac.uk/projects/seqmonk/

[80] Gasic K, Hernandez A, Korban SS (2004). RNA extraction from different apple tissues rich in polyphenols and polysaccharides for cDNA. Plant Mol Biol Report.

[81] Li H, Durbin R (2009). Fast and accurate short read alignment with Burrows-Wheeler transform. Bioinformatics.

[82] Li H, Handsaker B, Wysoker A, Fennell T, Ruan J, Homer N, Marth G, Abecasis G, Durbin R (2009). The sequence Alignment/Map format and SAMtools. Bioinformatics.

[83] Thompson JD, Higgins DG, Gibson TJ (1994). CLUSTAL W: improving the sensitivity of progressive multiple sequence alignment through sequence weighting, position-specific gap penalties and weight matrix choice. Nucleic Acids Res.

[84] Bandelt HJ, Forster P, Röhl A (1999). Median-joining networks for inferring intraspecific phylogenies. Mol Biol Evol.

[85] Paterson AH, Freeling M, Tang H, Wang X (2010). Insights from the comparison of plant genome sequences. Annu Rev Plant Biol.

