# Supplementary figures and images for: Building the sugarcane genome for biotechnology and identifying evolutionary trends
Source: BMC Genomics. 2014 Jun 30;15(1):540. doi: 10.1186/1471-2164-15-540 (PMC4122759; doi:10.1186/1471-2164-15-540)

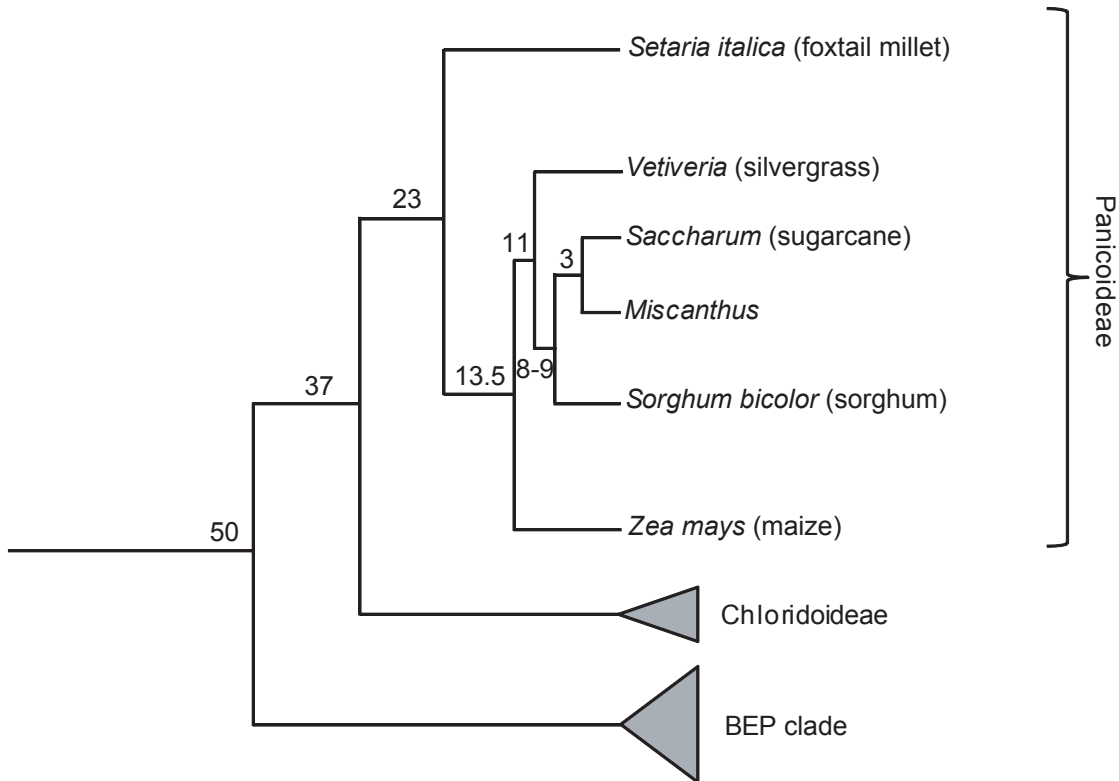

Supplement: Supplementary file 1 — Additional file 1: Figure S1: Schematic diagram of evolutionary history of grasses and sugarcane. BEP clade: Bambusoideae, Ehrhartoideae and Pooideae subfamilies. Numbers indicate divergence times [85]. (PDF 1 MB) [file 12864_2013_6311_MOESM1_ESM.pdf]

## A. Biological Processes

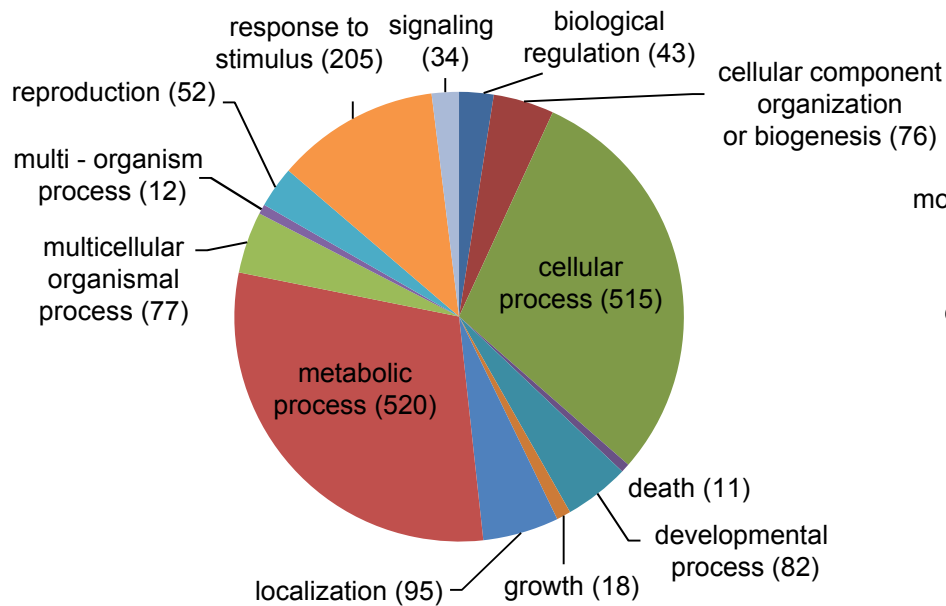

## B. Molecular Function

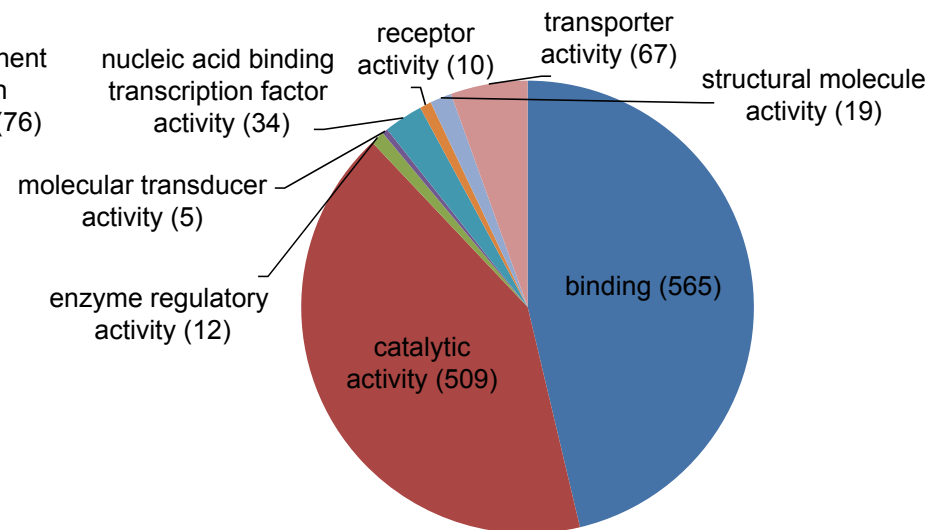

## C. Cellular Components

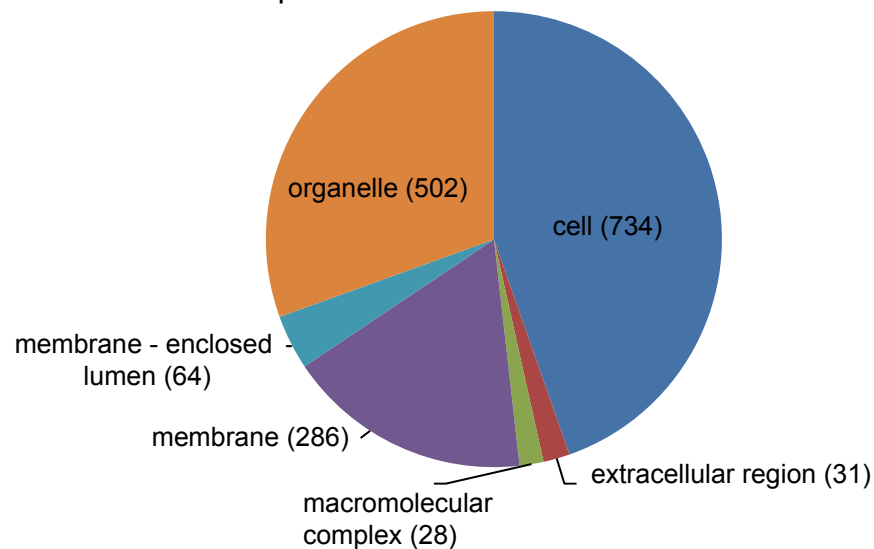

Supplement: Supplementary file 5 — Additional file 5: Figure S2: Distribution of Blast2GO annotations of protein-coding sequences. The chart shows level 2 annotations for A) Biological Processes, B) Molecular Function and C) Cellular Components. (PDF 144 KB) [file 12864_2013_6311_MOESM5_ESM.pdf]

# BLAST Top - Hits

Species

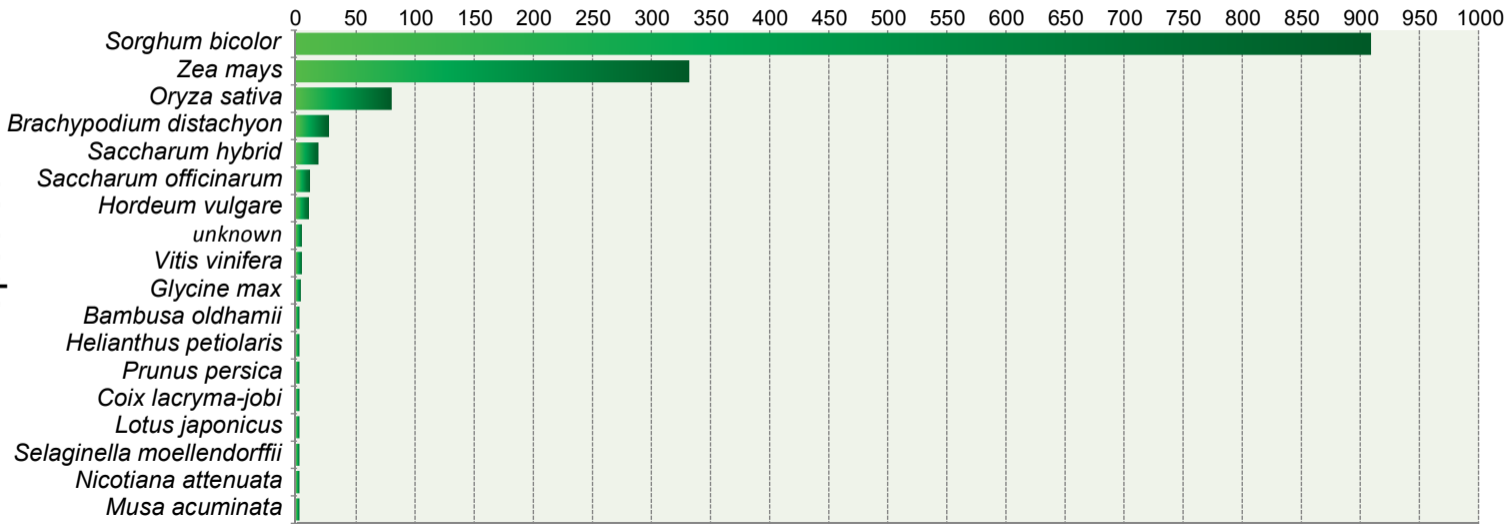

Supplement: Supplementary file 6 — Additional file 6: Figure S3: BLASTp best match distribution by species of the sugarcane putative protein-coding gene collection against the NCBI nr database. The species with the highest number of top-hits is S. bicolor, with 908 matches. (PDF 236 KB) [file 12864_2013_6311_MOESM6_ESM.pdf]

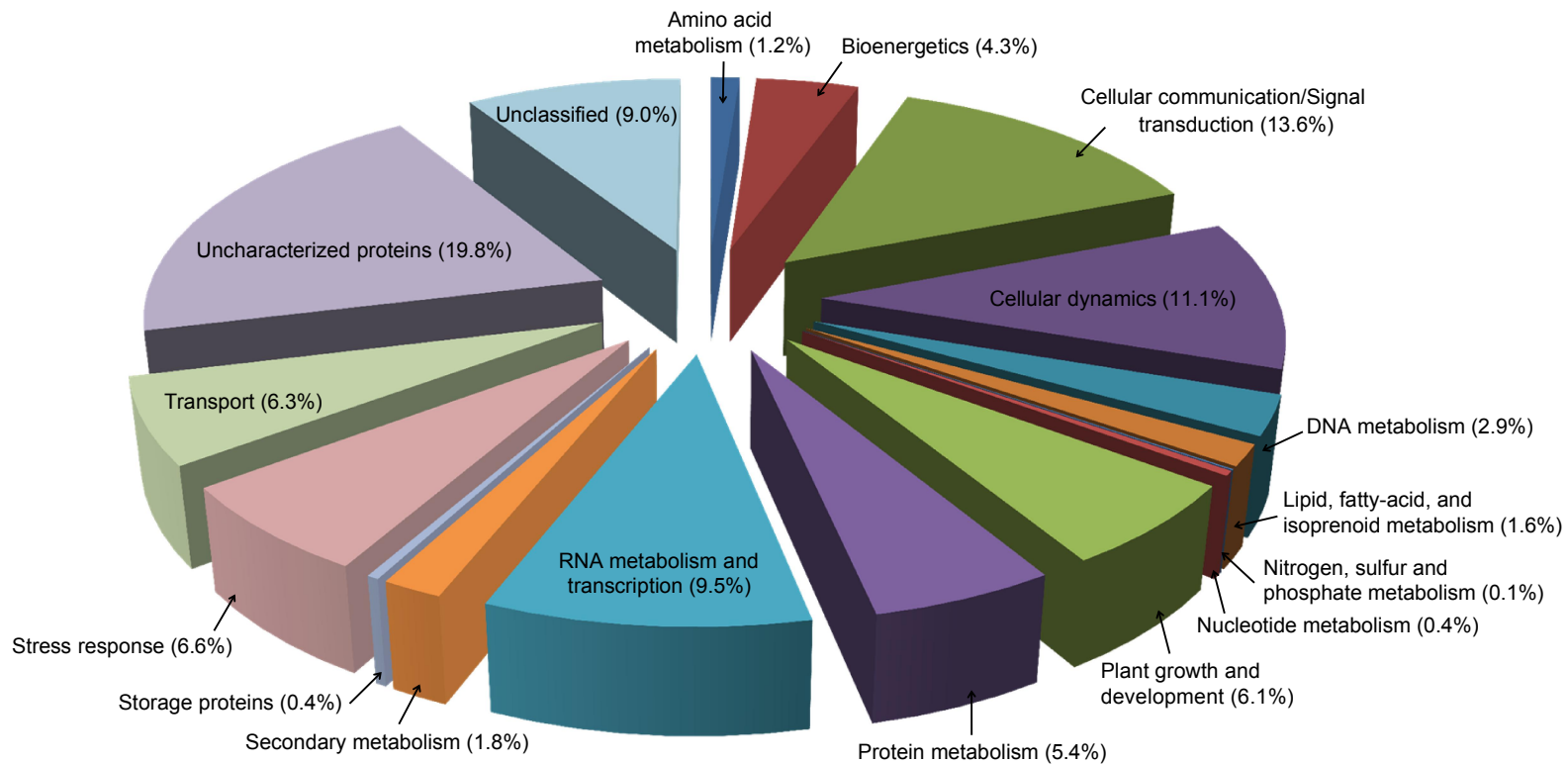

Supplement: Supplementary file 7 — Additional file 7: Figure S4: Annotation of sugarcane predicted protein-coding genes according to the 17 functional categories used in the sugarcane transcriptome study. The classification was done using the BLAST2GO tool (e-value < e-10). The 18th category “mobile genetic elements” proposed by Vettore et al. [8] was not included in this analysis since TE-derived genes were not included in the gene annotation. (PDF 502 KB) [file 12864_2013_6311_MOESM7_ESM.pdf]

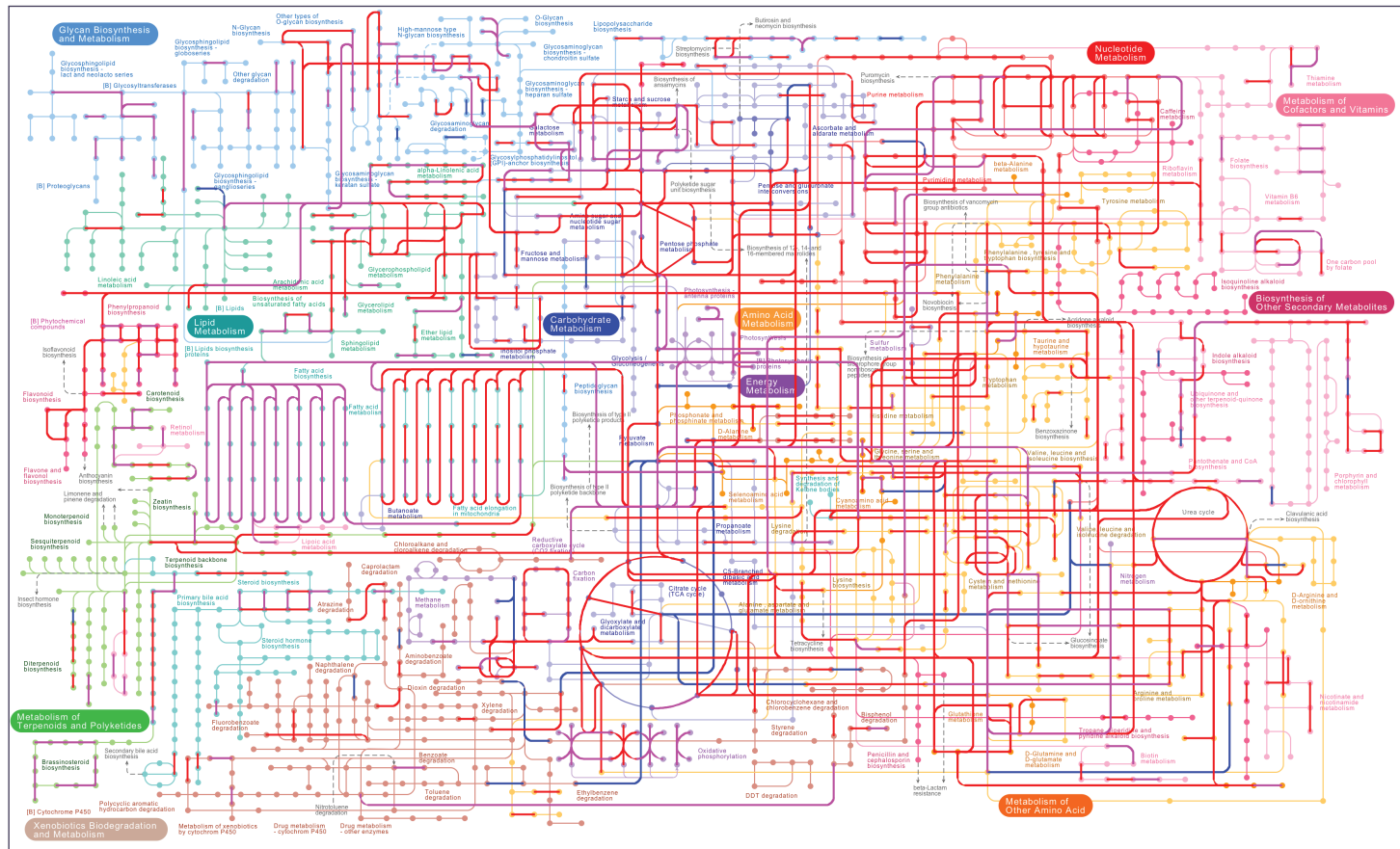

Supplement: Supplementary file 8 — Additional file 8: Figure S5: KeggMapper plot showing global metabolic pathways. Red lines indicate reactions for which predicted enzyme-coding genes were identified by sugarcane SASs, blue line indicate those identified by CDSs from sugarcane BACs and pink lines indicate those identified by both SASs and CDSs. Note that a single line may represent more than one match. (PDF 2 MB) [file 12864_2013_6311_MOESM8_ESM.pdf]

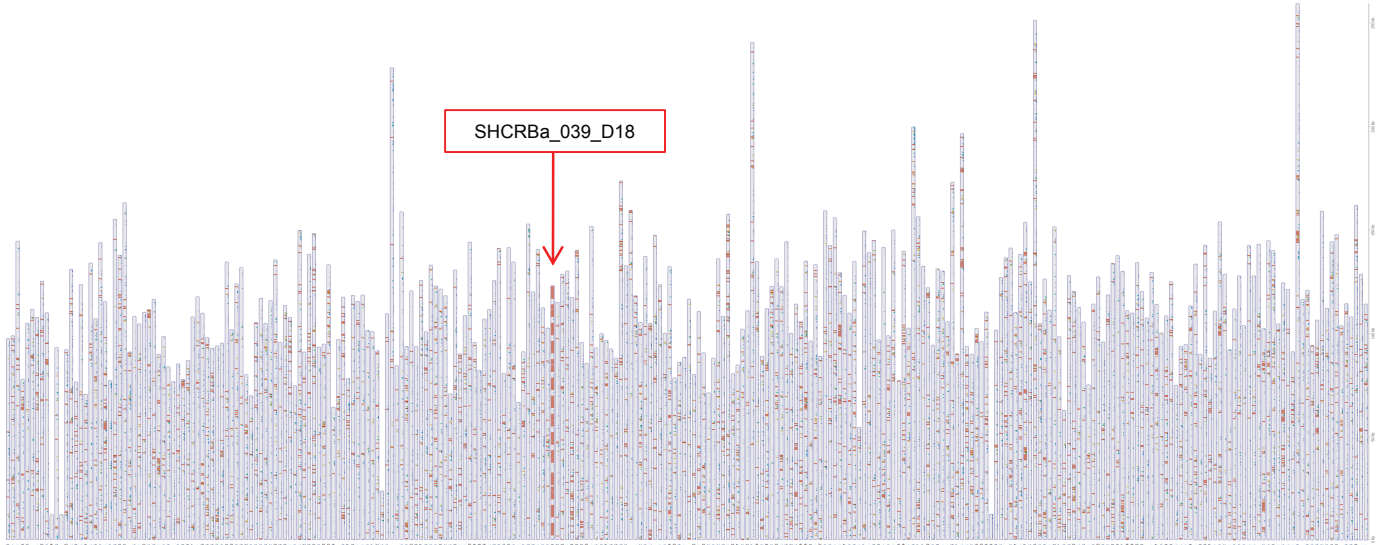

Supplement: Supplementary file 11 — Additional file 11: Figure S6: Global overview of sRNA mapping along sugarcane BACs. The horizontal colored bars shows extent of coverage. The colours of the bars are scaled from low (dark blue) to medium (green) to high (red). The rDNA BAC (SHCRBa_039_D18) has the highest number of sRNAs mapped. (PDF 4 MB) [file 12864_2013_6311_MOESM11_ESM.pdf]

A.

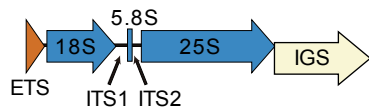

B.

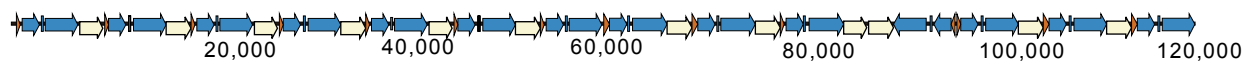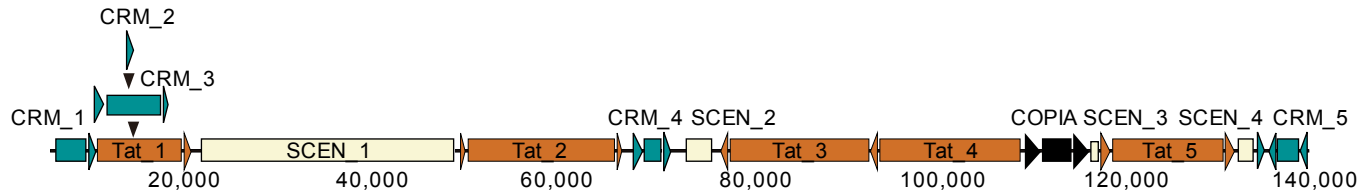

C.

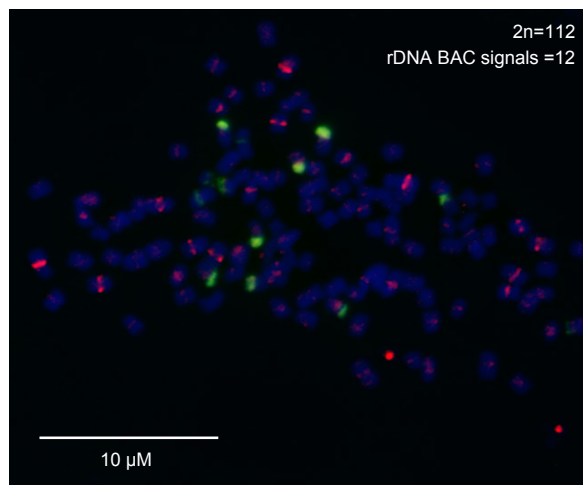

Supplement: Supplementary file 12 — Additional file 12: Figure S7: Structural organization and chromosomal location of the rDNA and the pericentromeric and/or centromeric BACs. A. Structure of the 45S ribosomal transcription unit identified in BAC (SCHRBa_039_D18). The BAC consists of 14 copies of the unit, one in reverse orientation to the other 13. ITS = internal transcribed spacer, ETS = external transcribed spacer, IGS = intergenic spacer. B. Simplified schematic of the centromeric BAC (SCHRBa_239_N21). The LTRs of the LTR retrotranspons are shown as arrows, the internal domains as squares. Each LTR retrotransposon is numbered consecutively. Black arrows indicate the location of the insertion of one element into another. ‘COPIA’ is an unidentified Copia-like element. C. Localization of the ribosomal (SCHRBa_039_D18) and pericentromeric/centromeric (SCHRBa_239_N21) BACs to metaphases from root tips of the sugarcane cultivar R570. Metaphases are counterstained with DAPI (blue). The centromeric BAC was detected with anti-digoxigenin-rhodamine (red), the ribosomal BAC with NeutrAvidin-Oregon Green-488 (green). (PDF 451 KB) [file 12864_2013_6311_MOESM12_ESM.pdf]

SHCRBa\_104\_G22

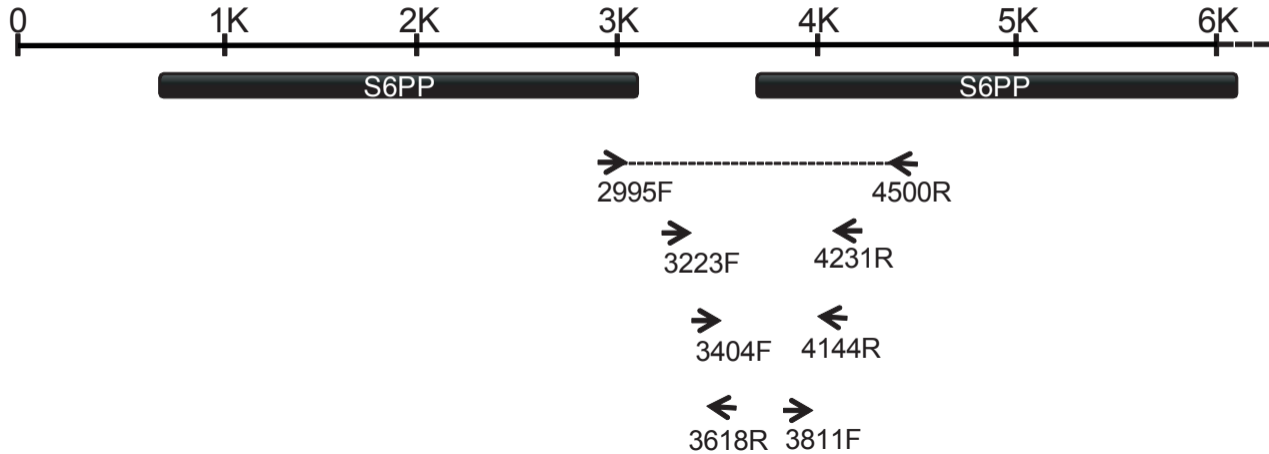

Supplement: Supplementary file 14 — Additional file 14: Figure S8: Location of the primers used to amplify the s6pp tandem gene duplication region. A 1539 bp fragment spanning the region was amplified from S. spontaneum, S. officinarum, modern sugarcane hybrid cultivars, Miscanthus sp. and sorghum using the primer pair 2995F and 4500R. All other primers are internal sequencing primers. Black bars indicate the two s6pp genes. 2995F: 5′ GCA GGG AGC GAG CAC ACG TT 3′, 4500R: 5′ TCG GTG CTC TCC CCT GCG AA 3′, 3223F: 5′ ACG ACC TTG CCT CTC TGT TG 3′, 4231R: 5′ TCA ACT TGT GAG GGA GAG CA 3′, 3404F: 5′ CAA TCG CTG TCG ATG GTG GC 3′, 4144R: 5′ CTG GCT GTA TCC GTA CAG AGG 3′, 3618R: 5′ AAG CTC TTG CCA GGA TTG CT 3′ and 3811F: 5′ GGC CGA GTT CTC CCA TGA TT 3′. (PDF 1 MB) [file 12864_2013_6311_MOESM14_ESM.pdf]
