# Supplementary material for: Building the sugarcane genome for biotechnology and identifying evolutionary trends
Source: BMC Genomics. 2014 Jun 30;15(1):540. doi: 10.1186/1471-2164-15-540 (PMC4122759; doi:10.1186/1471-2164-15-540)
Supplement: Supplementary file 4 — Additional file 4: Table S3: Summary of repeat content of sugarcane BACs. (PDF 184 KB) [file 12864_2013_6311_MOESM4_ESM.pdf]

Table S3. Summary of repeat content of sugarcane BACs.

	# Elements	Total length (Kb)	%
Retroelements	10234	13017.47	35.59
SINEs	66	7.95	0.02
Penelope	3	0.18	~ 0
LINEs	1465	712.18	1.95
R2/R4/NeSL	1	0.70	~ 0
RTE/Bov-B	262	168.76	0.46
L1/CIN4	1196	542.66	1.48
LTR elements	8703	12297.34	33.62
Ty1 / Copia	3034	5308.85	14.51
Gypsy / DIRS1	5548	6966.01	19.04
DNA transposons	12496	2901.88	7.93
hobo-Activator	1381	327.70	0.90
Tc1-IS630-Pogo	1883	323.28	0.88
En-Spm (CACTA elements)	2763	856.36	2.34
MuDR-IS905	1430	444.29	1.21
Tourist/Harbinger	2649	494.28	1.35
Rolling-circles	736	185.54	0.51
Unclassified	157	37.47	0.10
Total interspersed repeats (RM)	54007	16142.21	44.13
Additional sugarcane LTR-RT	-	1927.20	5.27
Total interspersed repeats	-	18069.41	49.40
Satellites	210	157.68	0.43
rDNA (SHCRBa_039_D18)	14	122.95	3.36
