# Supplementary material for: Building the sugarcane genome for biotechnology and identifying evolutionary trends
Source: BMC Genomics. 2014 Jun 30;15(1):540. doi: 10.1186/1471-2164-15-540 (PMC4122759; doi:10.1186/1471-2164-15-540)
Supplement: Supplementary file 10 — Additional file 10: Table S5: Detailed annotation of the sucrose and starch pathway genes and RNA-seq mapping. (PDF 187 KB) [file 12864_2013_6311_MOESM10_ESM.pdf]

Table S5. Detailed annotation of the sucrose and starch pathway genes and RNA-seq mapping.

EC number	Sugarcane gene	# RNA-seq reads mapped	Enzyme product
ec:2.4.1.12	SHCRBa_170_C07_R_370	6237	cellulose synthase
ec:2.4.1.13	SHCRBa_119_B13_R_140	1698	sucrose synthase
ec:2.4.1.13	SHCRBa_008_B11_R_90	918	sucrose synthase
ec:2.4.1.14	SHCRBa_014_G09_R_60	341	sucrose phosphate synthase
ec:2.4.1.14	SHCRBa_001_G20_F_90	235	sucrose phosphate synthase
ec:2.4.1.14	SHCRBa_063_N24_F_230	224	sucrose phosphate synthase
ec:2.4.1.14	SHCRBa_179_G22_F_90	207	sucrose phosphate synthase
ec:2.4.1.14	SHCRBa_181_K17_F_90	174	sucrose phosphate synthase
ec:2.4.1.18	SHCRBa_082_J13_F_70	3625	1,4-alpha-glucan branching enzyme
ec:2.4.1.21	SHCRBa_260_G18_F_100	547	starch synthase
ec:2.7.7.27	SHCRBa_026_K06_F_10	5065	glucose-1-phosphate adenylyltransferase
ec:2.7.7.27	SHCRBa_078_K12_F_350	3418	glucose-1-phosphate adenylyltransferase
ec:2.7.7.27	SHCRBa_003_M06_R_10	1295	glucose-1-phosphate adenylyltransferase
ec:2.7.7.27	SHCRBa_119_J13_R_150	501	glucose-1-phosphate adenylyltransferase
ec:2.7.7.27	SHCRBa_073_J10_R_160	448	glucose-1-phosphate adenylyltransferase
ec:2.7.7.27	SHCRBa_033_L20_R_100	448	glucose-1-phosphate adenylyltransferase
ec:2.7.7.27	SHCRBa_027_I16_R_250	443	glucose-1-phosphate adenylyltransferase
ec:2.7.7.27	SHCRBa_009_B01_R_20	79	glucose-1-phosphate adenylyltransferase
ec:2.7.7.27	SHCRBa_012_A01_R_60	63	glucose-1-phosphate adenylyltransferase
ec:2.7.7.27	SHCRBa_022_D05_R_200	22	glucose-1-phosphate adenylyltransferase
ec:2.7.7.27	SHCRBa_022_D05_R_110	4	glucose-1-phosphate adenylyltransferase
ec:2.7.7.9	SHCRBa_024_H17_F_300	2358	UDP glucose pyrophosphorylase
ec:3.1.1.11	SHCRBa_026_H12_R_50	24	pectinaesterase
ec:3.1.3.24	SHCRBa_104_G22_F_10	109	sucrose-6-phosphate phosphohydrolase
ec:3.2.1.15	SHCRBa_170_C07_F_90	23	polygalacturonase
ec:3.2.1.2	SHCRBa_185_C01_F_180	311	beta-amylase
ec:3.2.1.26	SHCRBa_119_J13_R_30	2	beta-fructofuranosidase
ec:3.2.1.37	SHCRBa_026_H12_F_40	1894	xylan 1,4-beta-xylosidase
ec:5.4.2.2	SHCRBa_018_K13_F_200	15413	phosphoglucomutase
